# Engineered Small Extracellular Vesicles Targeting Tumor‐Associated Endothelial Cells to Effectively Remodel the Glioma Microenvironment

**DOI:** 10.1002/advs.202518490

**Published:** 2026-01-21

**Authors:** Lingling Liu, Feiyang Xu, Zhiming Zheng, Xiaodan Yang, Fang Yang, Pei Liu, Yuankun Chen, Yunshu Yang, Junli Zhao, Peiyan Yang, Xiaojing Zheng, Xiaohong Sun, Ping Mao, Qinwen Mao, Hao Guan, Haibin Xia, Weifeng Zhang, Dan Xiao

**Affiliations:** ^1^ Department of Burns and Cutaneous Surgery Xijing Hospital Fourth Military Medical University Xi'an Shaanxi China; ^2^ Laboratory of Gene Therapy Department of Biochemistry College of Life Sciences Shaanxi Normal University Xi'an Shaanxi China; ^3^ Department of Neurosurgery Shandong Provincial Hospital Affiliated to Shandong First Medical University Jinan Shandong China; ^4^ Department of Neurosurgery The First Affiliated Hospital of Xi'an Jiaotong University Xi'an Shaanxi China; ^5^ Department of Pathology Huntsman Cancer Institute University of Utah Salt Lake City UT USA; ^6^ Department of Biochemistry and Molecular Biology Fourth Military Medical University Xi'an Shaanxi China

**Keywords:** glioma, immunotherapy, small extracellular vesicles, STING agonist, targeted therapy

## Abstract

Owing to the existence of the blood–brain barrier (BBB), achieving high‐efficacy, tumor‐selective delivery of therapeutic agents continues to be a pivotal unmet need in the treatment of glioblastoma. Here, based on the finding that CD93 is exclusively up‐regulated on glioma‐associated vascular endothelial cells (VECs), small extracellular vesicles (sEVs) were modified with insulin‐like growth factor‐binding protein 7 (IGFBP7), a natural ligand of CD93, to create a delivery platform that can deliver therapeutic agents to glioma‐associated VECs with high efficiency. At markedly reduced intravenous doses, IGFBP7‐sEVs efficiently concentrated temozolomide (TMZ) within glioma and elicited pronounced tumor growth inhibition. More strikingly, systemic administration of stimulator of interferon genes (STING) agonist‐loaded IGFBP7‐sEVs outperformed direct intratumoral injection of free STING agonist: the glioma microenvironment (GME) was extensively remodeled and antigen‐presenting capacity of myeloid cells was markedly enhanced. Moreover, endothelial‐restricted STING activation attenuated the exhaustion of CD8^+^ T cells. Consequently, the intensity of the tumor‐specific immune response was markedly augmented. Our data suggest that IGFBP7‐modified sEVs represent a novel platform that enables highly efficient, glioma VECs‐targeted delivery of therapeutics into glioma, and are adaptable to a broad spectrum of agents, especially immunomodulators. It is a novel and effective strategy for treating gliomas.

## Graphical abstract

This study developed a novel drug delivery platform that overcomes the blood‐brain barrier in glioblastoma. By fusing IGFBP7 with small extracellular vesicles, the platform specifically targets tumor vasculature. It effectively delivers temozolomide, suppressing tumor growth at low doses. Moreover, when loaded with immune agonists, it remodels the tumor microenvironment and activates robust anti‐tumor immunity, presenting an innovative and versatile strategy for glioma therapy.

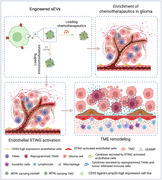

## 1 Introduction

Glioma is the most common primary brain tumor, characterized by high malignancy and poor prognosis. Currently, the standard clinical approach for glioma therapy involves surgical resection combined with radiotherapy and chemotherapy. However, recurrence after surgery is almost inevitable. The 5‐year survival rate for patients with glioblastoma is typically less than 5% [1, 2, 3, 4]. The difficulty in treating glioma is caused by multiple reasons, one of the most challenging problems is efficient drug delivery [3, 5, 6, 7, 8, 9, 10, 11]. Owing to the presence of the blood‐brain barrier (BBB), peripheral delivered drugs are difficult to reach glioma [12, 13, 14, 15]. Meanwhile, intracranial drug delivery is associated with high surgical difficulty and risk. Therefore, it is of great significance to develop new drug delivery strategies for glioma therapy.

Due to their unique anatomical position, vascular endothelial cells (VECs) located in the glioma microenvironment (GME) are an ideal target for glioma drug delivery. First, targeting glioma VECs eliminates the need to cross the BBB, thereby improving drug delivery efficiency. Second, glioma VECs are located at the interface between the central nervous system (CNS) and the peripheral immune system, which endows them crucial role in the communication between peripheral immune responses and CNS immune response [16, 17, 18, 19, 20]. Studies have shown that under the long‐term domestication of the tumor microenvironment (TME), glioma VECs exhibit different expression profile characteristics from other VECs [21, 22, 23]. Among them, CD93 is exclusively upregulated in glioma VECs, suggesting its potential as a target molecule for drug delivery to glioma VECs [24]. Here, we hope to modify small extracellular vesicles (sEVs) to target the CD93 molecules on glioma VECs, thereby using them as carriers for drug delivery to glioma. For chemotherapy, this method allows chemical drugs to be enriched in the TME, thereby enhancing their efficacy and safety. More importantly, delivery of immunomodulators to glioma‐associated endothelial cells can specifically activate glioma VECs, leveraging the gatekeeper function of VECs to synchronize systemic and intracranial immune responses, thereby amplifying intratumoral inflammation and remodeling TME.

## 2 Results

### 2.1 Expression of CD93 in Glioma VECs

**FIGURE 1:**
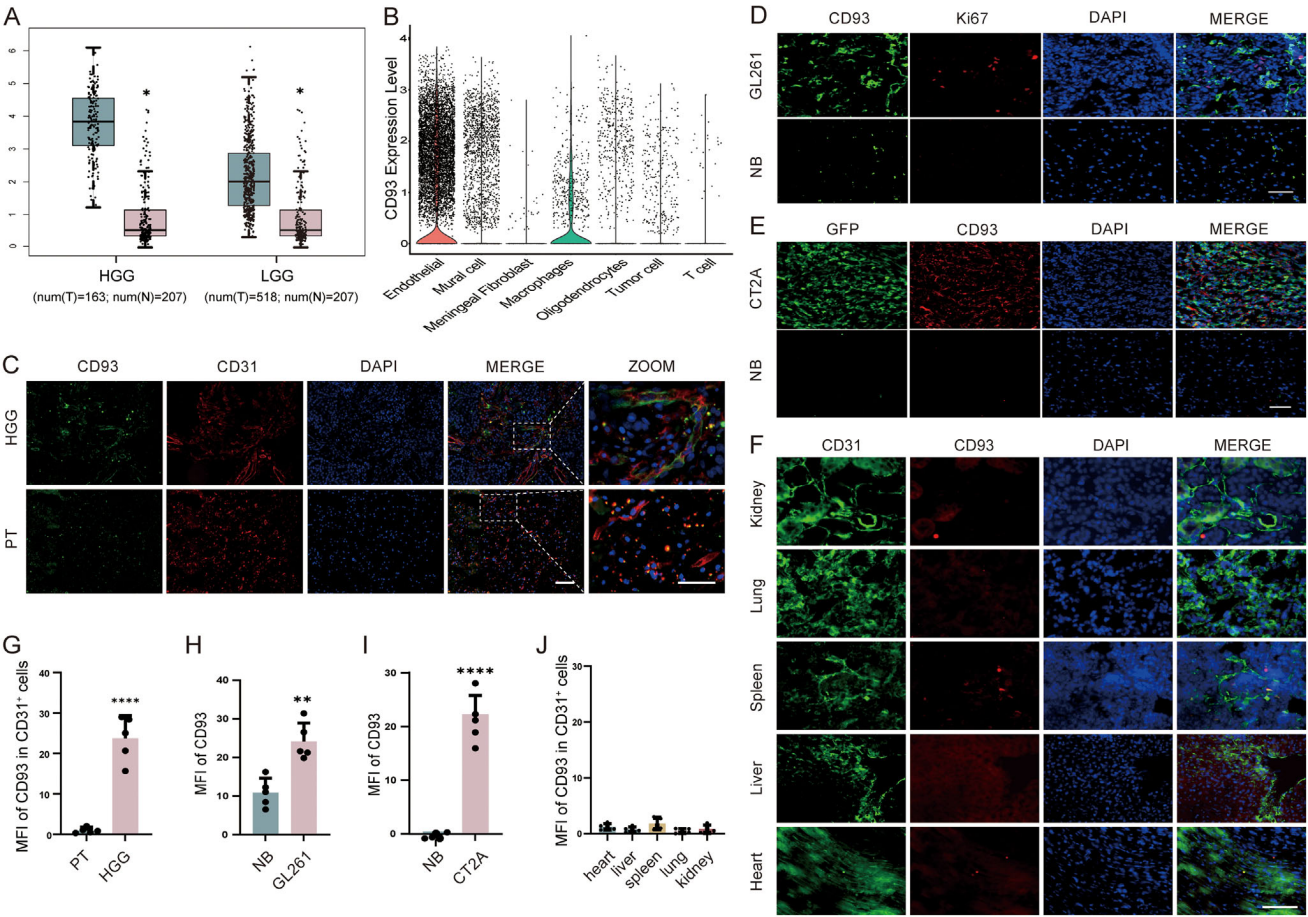
Validation of CD93 Expression in Glioma Vecs. (A‐B) Bioinformatic analysis of CD93 in different type of gliomas (A) and different cells in glioma (B). (C) Expression of CD93 in human glioblastoma sample, representative of five individual replicates (PT, peritumoral tissue). Scale bar=50 µm, Scale bar of zoomed image: 25 µm. (D) Expression of CD93 in GL261 mouse glioma tissue, representative of five individual replicates (NB, normal brain). Scale bar=50 µm. (E) Expression of CD93 in CT2A‐GFP mouse glioma tissue, representative of five individual replicates (NB, normal brain). Scale bar=50 µm. GFP positive cells indicate tumor cells. (F) Expression of CD93 in mouse peripheral organs, representative of five individual replicates. Scale bar=25 µm. (G‐J) Statistic analysis of C‐F, n=5 per group. G‐I, unpaired two‐tailed t test. J, one‐way ANOVA, All results are expressed as mean ± SD, ^*^ *p* < 0.05, ^**^ *p* < 0.01, ^*^^***^ *p* < 0.0001.

CD93 was previously identified as one of the top‐ranked genes involved in tumor angiogenesis, consistently overexpressed among head and neck squamous cell carcinomas (HNSCCs), breast cancers (BCs), and clear cell renal cell carcinomas (CCRCCs) [25]. What's more, CD93 overexpression has been observed in the vasculatures of multiple other tumors, including glioma [26, 27, 28]. The overexpression of CD93 may be due to VEGF exposure in TME [29], blockade of VEGF signaling by an antibody can reduce CD93‐positive cells [25]. We analyzed the single‐cell RNA sequencing (scRNA‐seq) results from public databases and verified the increased expression of CD93 in the vasculature of high‐grade gliomas (HGG) and low‐grade gliomas (LGG) (Figure 1A,B). This result was further confirmed by immunofluorescence staining of glioma tissues from patients and mouse model (Figure 1C–E,G–I; Figure S1). The expression of CD93 was significantly elevated in the VECs within glioma, as compared to that in normal tissues (Figure 1C,F,G,J; Figures S1 and S2). Specifically, in the GL261 glioma model, CD93 is enriched 7.5‐fold on glioma endothelium relative to normal brain endothelium and 13∼45‐fold relative to the vasculature of peripheral tissues. These results demonstrate that CD93 was selectively and markedly up‐regulated in glioma VECs, and can be used as a target molecule for drug delivery to the vasculature in gliomas.

### 2.2 sEVs Modification to Improve Glioma VECs Targeted Delivering Efficiency

**FIGURE 2:**
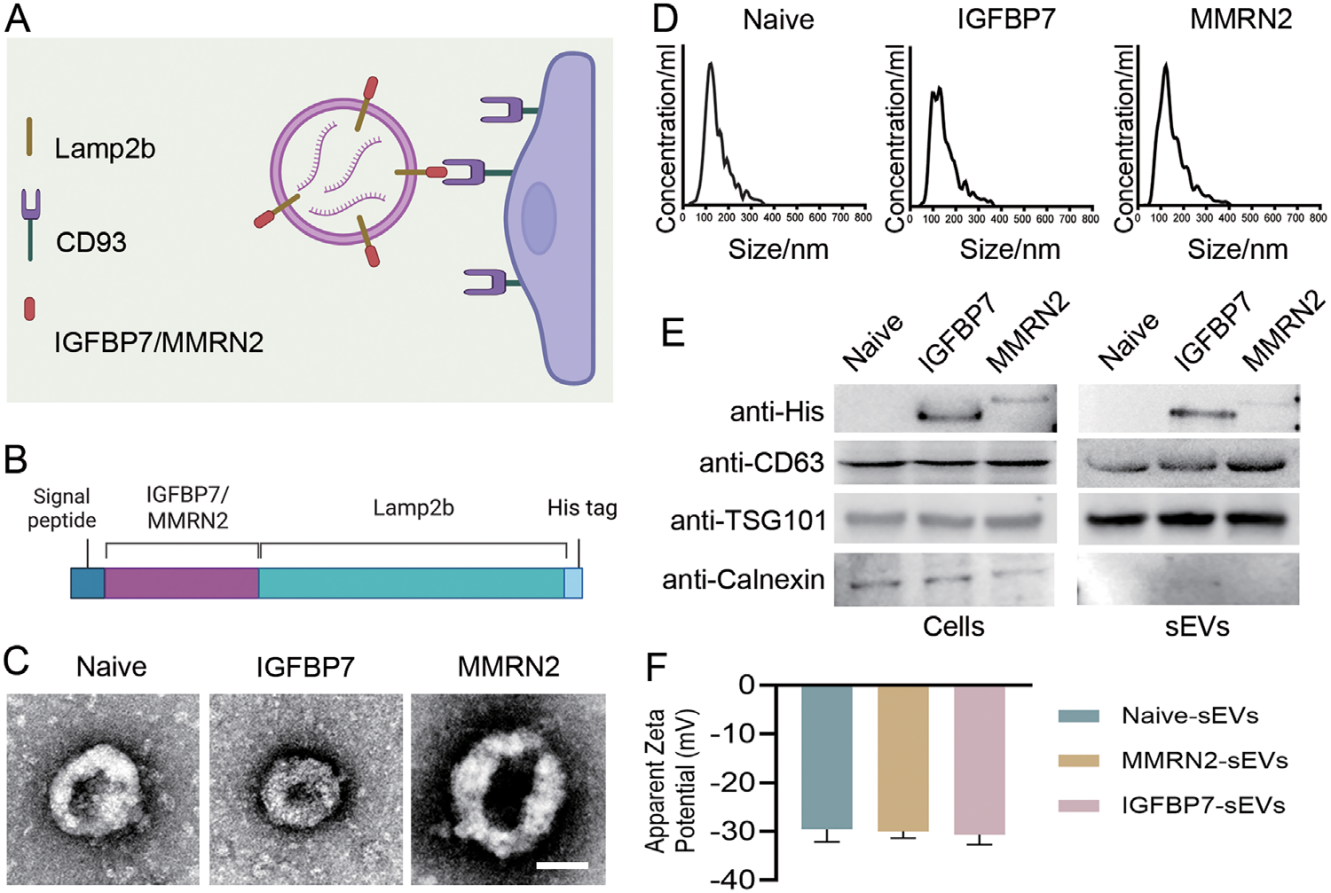
Modification and Qualification of sEVs. (A) Schematic diagram of sEVs modification principle. (B) Plasmid structures for the indicated Lamp2b‐ligand fusion proteins. (C) sEVs electron microscopy images. Scale bar=100 nm. (D) Nanoparticle tracking analysis (NTA) of the particle size of indicated sEVs. (E) Western blot analysis of CD93 ligand loading in sEVs. One representative of 3 experiments is shown in C, D, E. (F) Surface charge of sEVs.

sEVs, also known as nanosomes, are vesicles secreted by cells with sizes ranging from 30 to 150 nm. They can carry nucleic acids, proteins, and other substances from cells and deliver them to other cells. By modifying the membrane proteins of sEVs, their delivery efficiency to specific cells can be specifically enhanced [30]. Therefore, sEVs are ideal drug delivery carriers. Multimerin‐2 (MMRN2) and insulin‐like growth factor‐binding protein 7 (IGFBP7) have been reported as ligands for CD93 [31]. Therefore, we plan to enhance the delivering efficiency of sEVs to glioma VECs by modifying Lamp2b, one of the most exosomal transmembrane proteins, with MMRN2 or IGFBP7 (Figure 2A). MMRN2 or IGFBP7 was fused to the N terminus of Lamp2b as reported [32] to make it exposed to the surface of sEVs. His tag was inserted into the C terminus of Lamp2b for the detection of MMRN2 or IGFBP7 loading on sEVs (Figure 2B). MMRN2‐Lamp2b and IGFBP7‐Lamp2b modified sEVs are physically similar with naïve sEVs as analyzed by transmission electron microscopy (TEM) (Figure 2C; Figure S3A) and nanoparticle tracking analysis (NTA) (Figure 2D), indicating that the modification of Lamp2b with MMRN2 or IGFBP7 did not change the characteristics of the sEVs. Western blot assay verified that both fusion proteins were expressed in the cell lines and the derived sEVs, but the expression of IGFBP7‐Lamp2b was significantly higher than MMRN2‐Lamp2b both in the cell lines and the derived sEVs (Figure 2E; Figure S3B–E). sEVs exhibited a negative surface charge of ∼ −30 (zeta potential) (Figure 2F), indicating a stable colloidal suspension. Since the expression of MMRN2‐Lamp2b in the cell lines is significantly lower than that of IGFBP7‐Lamp2b, we wonder whether the modification by MMRN2 affected the transfection efficiency of the plasmid or the expression level of the MMRN2‐Lamp2b fusion gene. Therefore, we co‐transfected the two modified plasmids with a plasmid carrying the GFP reporter gene into HEK293 cells and detected differences in transfection efficiency and gene transcription levels, respectively. The result shows that although the transfection efficiency of MMRN2‐Lamp2b is slightly lower than that of IGFBP7‐Lamp2b (Figure S4A), the difference is not significant, indicating that transfection efficiency is not the main reason for the difference in protein levels. The reverse transcription polymerase chain reaction (RT‐PCR) result shows that the transcription level of MMRN2‐Lamp2b is only half that of IGFBP7‐Lamp2b (Figure S4B), suggesting that the difference in protein levels might be due to the effect of MMRN2 modification on the transcription of the fusion gene.

**FIGURE 3:**
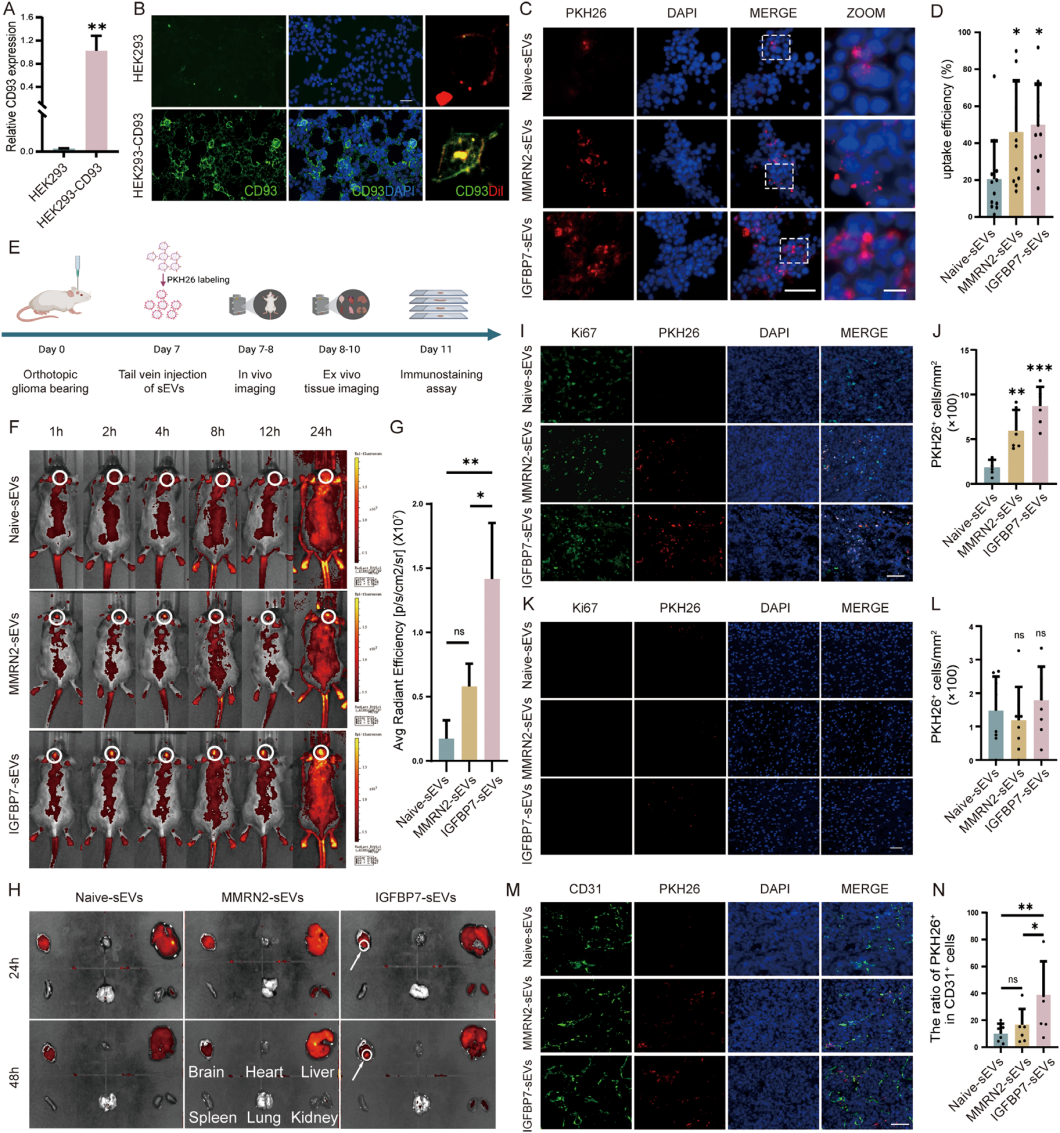
Analysis of sEVs Uptake Efficiency after Modification. (A‐B) Analysis of CD93 expression in HEK293‐CD93 cell line. HEK293 cells were infected by lentivirus carrying CD93 gene, and the expression of CD93 was analyzed by RT‐PCR (A) and immunofluorescence staining (B). A, n=3 per group, unpaired two‐tailed t test. B, Representative of three individual replicates, scale bar=50 µm. (C) Immunostaining analysis of the uptake efficiency of modified sEVs in vitro. Indicated sEVs were labeled with PKH26 dye and incubated with HEK293‐CD93 cell line for 24 h, then cells were washed by PBS for 3 times and observed under fluorescence microscope. The dashed white box indicates the zoomed region. Four fields were randomly selected from each sample. Representative of three individual replicates. Scale bar=50 µm. (D) Quantification of PKH26 positive cells. n=3 independent samples, one‐way ANOVA was performed to determine the statistical significance. (E) Schematic diagram of in vivo uptake efficiency assay. (F) Representative images of sEVs localization in different treatment groups. The distribution of sEVs was monitored using IVIS imaging system at indicated time points post sEVs injection. The white circles indicate the sEVs that have accumulated within the tumor region. Representative of three individual replicates. (G) Quantitative analysis of PKH26 fluorescence in the tumor at 24 h post sEVs injection. n=3 mice per group, one‐way ANOVA. (H) Ex vivo imaging to analyze the distribution of sEVs in different tissues. Mice were sacrificed at 24 or 48 h post sEVs injection, tissues were removed and detected using IVIS imaging system. The white circles indicate the accumulated sEVs within the tumor region. Representative of three individual replicates. (I,K) Immunofluorescence evaluation of localization of sEVs (PKH26, red) within the tumor area (Ki67^+^, green) (I) and non‐tumor area (Ki67‐) (K). Representative of five individual replicates. Scale bar=50 µm. (J,L) Quantitative analysis of PKH26 positive cells in tumor area (J) and non‐tumor area (L). n=5 mice per group, one‐way ANOVA. (M) Immunofluorescence evaluation of co‐localization of sEVs (PKH26, red) and VECs (CD31, green). Representative of five individual replicates. Scale bar=50 µm. (N) Quantification of the percentage of PKH26 positive VECs in the tumor region. The number of CD31‐positive cells and the number of PKH26‐positive cells among the CD31‐positive cells were quantified, and the percentage of PKH26‐positive cells relative to the CD31‐positive cells was calculated. n=5 mice per group, one‐way ANOVA. All results are expressed as mean ± SD, ^*^ *p* < 0.05, ^**^ *p* < 0.01, ^***^ *p* < 0.001, ns=not significant.

Next, we analyzed the delivery efficiency of modified sEVs in vitro and in vivo. First, a CD93 overexpression cell line was built by transduction of HEK293 cells with CD93 expression lentivirus. The overexpression of CD93 was verified by RT‐PCR (Figure 3A). Immunofluorescence staining further confirmed that a large amount of CD93 was localized on the cell membrane (Figure 3B). Then, CD93 overexpression cells were incubated with PKH26 labeled sEVs, the results indicated that IGFBP7‐ and MMRN2‐modified sEVs exhibited enhanced uptake into CD93‐negative HEK293 cells compared with naïve sEVs (Figure 3C,D), whereas their uptake into unmodified HEK293 cells did not differ (Figure S5). These results indicate that modification of sEVs with IGFBP7 and MMRN2 can enhance their uptake via CD93 mediated internalization. As we had verified, both GL261 and CT2A mouse glioma models displayed exclusive CD93 expression in VECs in GME (Figure 1C–J; Figures S1 and S2). To validate whether modifying sEVs enhances their uptake by glioma VECs, we conducted in vivo experiment using a GL261 tumor‐bearing model (Figure 3E). After intravenous injection of PKH26‐labeled sEVs, aggregation of fluorescent signals can be detected in the brains of mice injected with the modified sEVs, while no such aggregation of fluorescent signals was observed in the brains of mice injected with the naïve sEVs (Figure 3F–H). As expected, because of the filtering effect of the liver on venous blood, a strong fluorescence signal can also be detected in the livers of mice injected with different sEVs (Figure 3H; Figure S6). Next, mouse tissues were subjected to immunofluorescence staining, and the mean fluorescence intensity of PKH26 was quantified to analyze the distribution of PKH26‐labeled sEVs in vivo. For mice injected with IGFBP7‐modified sEVs, the tumor displayed the highest fluorescence intensity, confirming selective enrichment of the engineered sEVs at the neoplastic site. In contrast, fluorescence in normal brain and all peripheral organs—except the liver—remained very low (Figure 3I–L; Figure S7). A pronounced signal was detected in the liver, consistent with the ex vivo findings (Figure S7). Collectively, these findings indicate that although intravenously administered modified sEVs exhibit some off‐target distribution, the impact on most tissues is minimal. In the liver, PKH26 fluorescence predominantly colocalizes with VECs, although a fraction is also observed within macrophages. Notably, the hepatic retention of IGFBP7‐modified sEVs appears to be reduced compared with naïve sEVs and MMRN2‐modified sEVs (Figure S7). The density of PKH26‐positive cells per unit area within the tumor was three‐ to fourfold higher in mice that received engineered sEVs than in those given naive sEVs, with IGFBP7‐modified sEVs showing the most pronounced intratumoral accumulation (Figure 3I,J). In contrast, the density of PKH26‐positive cells in normal brain parenchyma remained low and did not differ significantly between engineered and naive sEVs (Figure 3K,L). Meanwhile, co‐staining of CD31 with PKH26 reveals that PKH26 was mainly localized within VECs. IGFBP7‐modified sEVs transduced ∼40 % of intra‐tumoral CD31^+^ cells, whereas naive sEVs reached only ∼10 % (Figure 3M,N). In contrast, fewer than 6 % of intratumoral CD31^−^ cells contain sEVs signal (Figure S8). These results further demonstrate that the modified sEVs can specifically enhance the delivery efficiency to glioma VECs after intravenous injection, while IGFBP7‐modified sEVs have the highest delivery efficiency. So, IGFBP7‐modified sEVs were selected for further in vivo therapeutic studies.

### 2.3 Delivery of TMZ by Modified sEVs Improves the Therapeutic Effect

Temozolomide (TMZ) is a derivative of dacarbazine and is the most commonly used chemotherapeutic drug in the clinical treatment of gliomas [33]. Although TMZ can cross the BBBs to reach tumor tissues, it has multiple limitations, such as short half‐life (about 2 h), rapid clearance, and low tissue targeting, which resulted in lesser brain accumulation [34]. Therefore, a relatively high drug concentration is required to achieve an anti‐tumor effect, which can easily lead to side effects. Approximately 15% of patients discontinue treatment due to the side effects of TMZ therapy [33]. If TMZ could be efficiently delivered to glioma VECs via sEVs, it would provide many advantages, such as improving the stability of TMZ and enhancing the delivering efficiency to GME. As a result, it would be possible to achieve a high local drug concentration in the GME with low systemic drug administration, improving the spread of TMZ to tumor cells. This approach not only enhances the safety of the treatment but also maximizes the drug concentration in gliomas while maintaining safe systemic drug doses, thereby achieving superior therapeutic outcomes.

**FIGURE 4:**
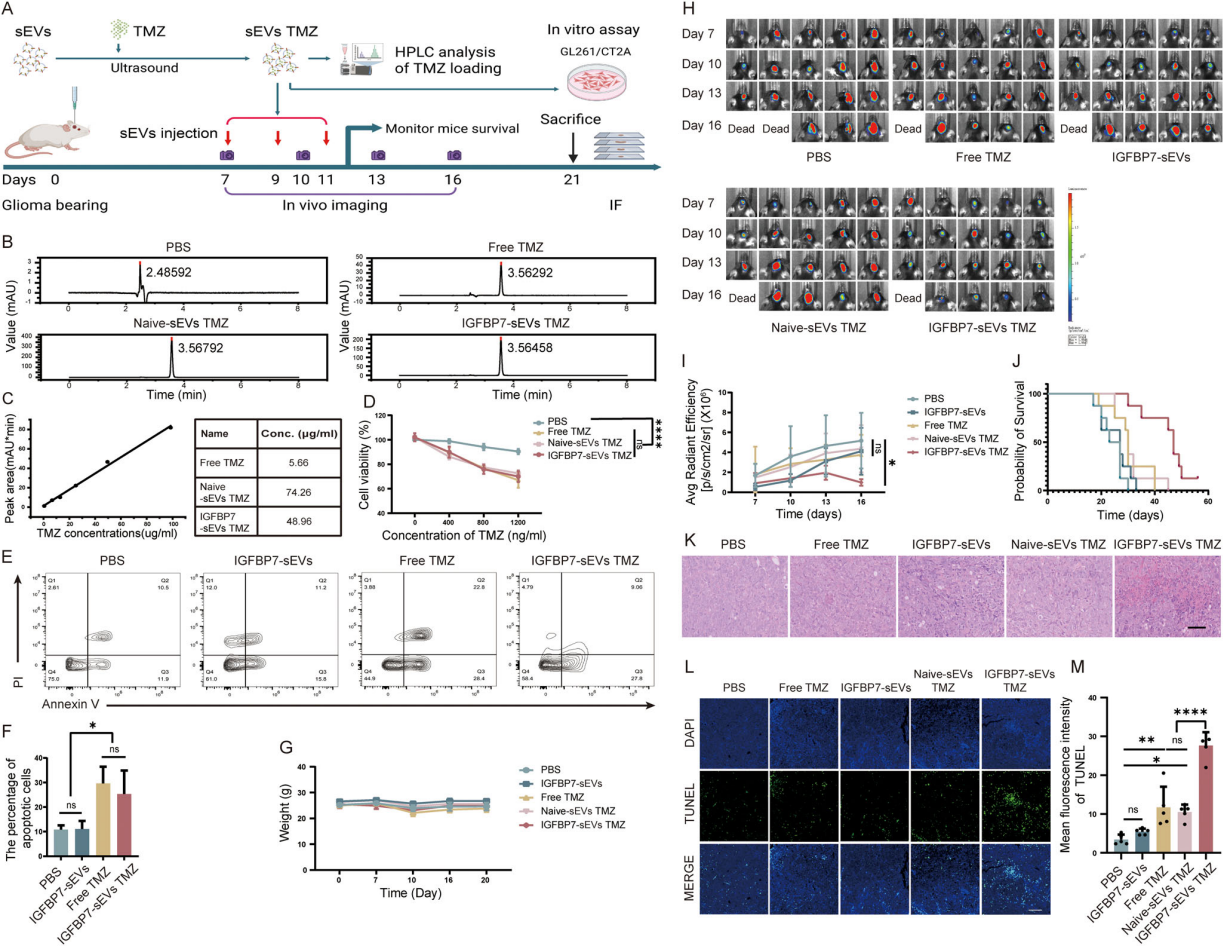
TMZ Loaded in IGFBP7‐Modified sEVs Exhibits Glioma Inhibition Effect after i.v. Injection. (A) Schematic diagram of experimental timeline. (B,C) HPLC analysis of TMZ loading efficiency. IGFBP7 modified sEVs were collected and loaded with TMZ through ultrasound. Loading efficiency was analyzed by HPLC. (D) CCK‐8 evaluates the survival of GL261 cells after treated with free TMZ or TMZ loaded sEVs with indicated doses, the survival of cells was analyzed 48 h post treatment. n=3 independent samples per group, one‐way ANOVA. (E) Flow cytometry analysis of cell apoptosis. GL261 cells were treated with 100 µg/ml free TMZ or sEVs loaded with the same dose of TMZ, the apoptosis of cells were analyzed 48 h post treatment. Representative of three individual replicates. (F) Statistic analysis of (E). n=3 independent samples per group, one‐way ANOVA. (G‐M) GL261 cells were implanted into the striatal region of C57 mice to establish an orthotopic glioma model, drug loaded sEVs were injected through tail vein 7 days after tumor cells implantation every two days for three times consecutively. The volume of tumors was monitored at indicated time points. (G) Mouse weight change curve. (H) In vivo imaging of tumor volume. Tumor volumes were monitored every 3 days from 7 days post tumor cell inoculation. (I) Tumor growth curve. n=5 mice per group, one‐way ANOVA. (J) Mice survival curve. n=8 mice per group, Kaplan‐Meier. (K) Histological staining analysis of tumor tissue. Scale bar=100 µm. (L) TUNEL stain analysis of tumor cell apoptosis. Scale bar=100 µm. (M) Quantification of TUNEL positive cells. Four fields were randomly selected from each mouse. n=5 mice per group, one‐way ANOVA. All results are expressed as mean ± SD, ^*^ *p* < 0.05, ^**^ *p* < 0.01, ^****^ *p* < 0.0001, ns=not significant.

Thus, we verified the therapeutic effect of delivering TMZ to glioma VECs via modified sEVs (Figure 4A). TMZ was loaded into sEVs using ultrasound, and the drug‐loading efficiency was analyzed by high‐performance liquid chromatography (HPLC) (Figure 4B,C). First, GL261 cells were treated with both free TMZ and TMZ‐loaded sEVs in vitro. The results showed that, at the same TMZ dosage, TMZ‐loaded sEVs and free TMZ had similar inhibitory effects on tumor growth and similar promoting effects on tumor cell apoptosis (Figure 4D–F). These results further confirmed the accuracy of the TMZ‐loading quantification results and also indicated that encapsulating TMZ in sEVs did not affect its cytotoxicity. Next, we used an orthotopic mouse glioma model to test the in vivo therapeutic effect of TMZ‐loaded sEVs (Figure 4A). The body weight change curves of mice showed that none of the treatments affected the growth of mice (Figure 4G). Mice injected with TMZ‐loaded IGFBP7‐sEVs exhibited significant tumor growth inhibition, whereas those injected with the same dose of free TMZ or TMZ‐loaded naïve sEVs had tumor growth curves similar to the PBS‐injected group (Figure 4H,I). This indicates that IGFBP7 modification enhanced the accumulation of sEVs in the GME, effectively increasing the local drug concentration in the tumor under low‐dose intravenous TMZ administration, thereby achieving better anti‐tumor effects compared with TMZ‐loaded naïve sEVs or free TMZ. Additionally, the tumor growth curve of the group injected with naïve sEVs (without TMZ loading) did not change significantly compared with the PBS‐injected group (Figure 4I). Some studies have shown that the interaction between IGFBP7 and CD93 on tumor VECs is one of the causes of abnormal tumor vasculature. Blocking the binding of IGFBP7 and CD93 can promote the normalization of tumor vasculature and produce anti‐tumor effects [29]. Theoretically, IGFBP7 on the surface of modified sEVs membranes could also bind to CD93, exacerbating the abnormality of tumor vasculature and thus negatively affecting the therapeutic effect of drug‐loaded sEVs. However, the above results revealed that IGFBP7‐modified sEVs did not affect glioma growth directly. Probably because IGFBP7 has already been highly expressed in the TME [29, 31], so the IGFBP7 on the surface of modified sEVs cannot further enhance the activation of CD93. TMZ‐loaded IGFBP7‐sEVs treatment also significantly improved the survival of glioma‐bearing mice (Figure 4J). Hematoxylin and eosin (HE) and TdT‐mediated dUTP Nick End Labeling (TUNEL) stain revealed extensive tumor cell death in IGFBP7‐sEVs TMZ‐treated mice (Figure 4K–M). To verify the safety of different treatments, the peripheral tissues of mice with different treatments were subjected to histological staining. The results indicated that none of the treatments caused damage to the peripheral tissues (Figure S9). These results indicate that IGFBP7‐modified sEVs can be used to deliver chemotherapeutic drugs to glioma VECs and locally increase the concentration of drugs in the tumor, thereby enhancing therapeutic efficacy and safety.

### 2.4 Delivery of STING Agonist by IGFBP7‐Modified sEVs Efficiently Suppresses Tumor Growth

**FIGURE 5:**
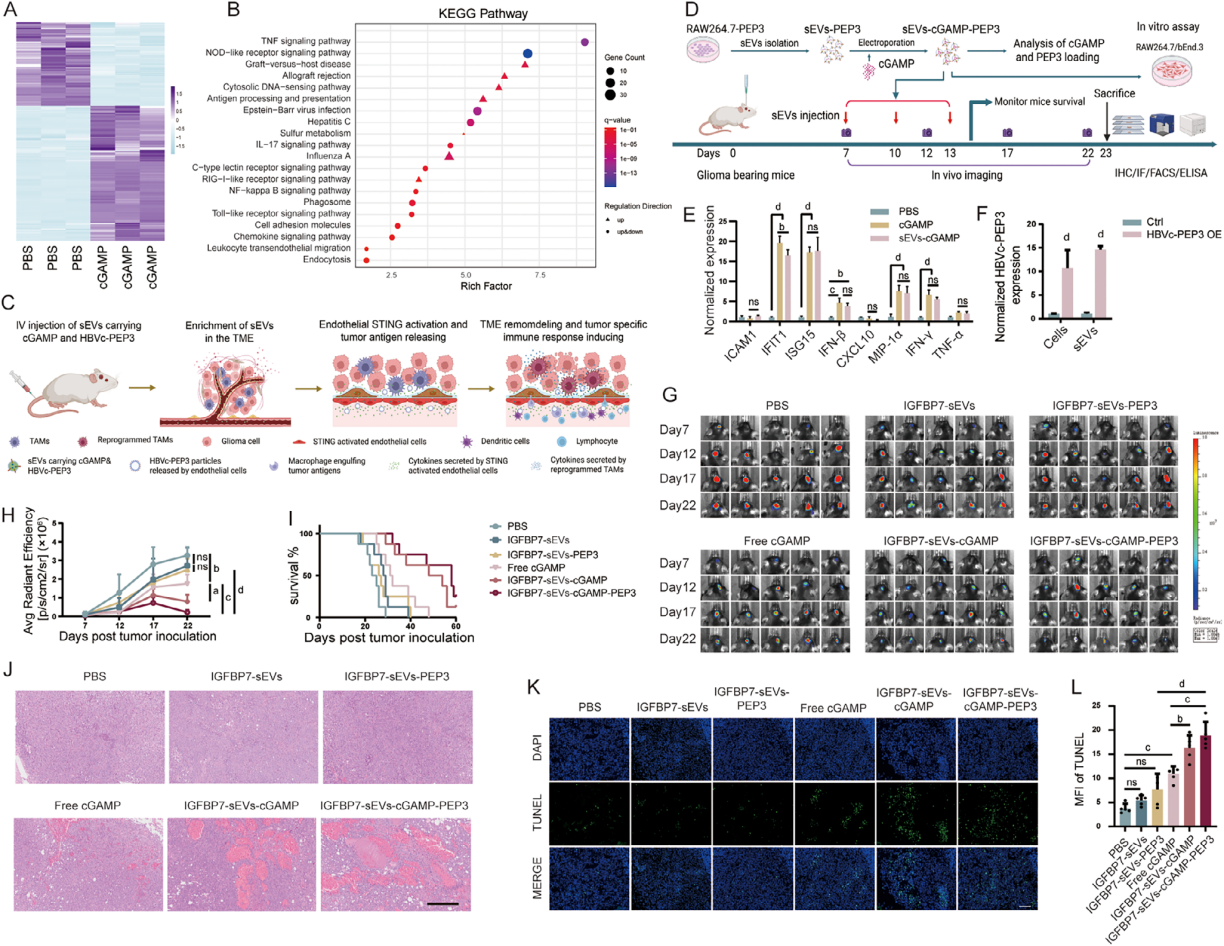
I.v. Injection of cGAMP Loaded sEVs Suppresses Glioma. (A, B) RNA seq analysis of the expression profile changes in vascular endothelial cells after STING activation. bEnd.3 cells were stimulated with 20 µg/mL cGAMP for 48 h, cells were collected and analyzed by next generation sequencing. (A) Heatmap reveals the changes of expression profile after STING activation. (B) KEGG enrichment analysis. (C) Cartoon shows the hypothesis of sEVs loaded with STING agonist and tumor antigen mediated immunotherapy. (D) Schematic diagram of experiment timeline. (E) In vitro comparation of STING activation ability between free cGAMP and cGAMP loaded sEVs. bEnd.3 cell line was stimulated with 10 µg/mL free cGAMP or IGFBP7 modified sEVs carrying the same doses of cGAMP for 48 h, the expression of STING downstream molecules was analyzed by RT‐PCR. n=3 independent samples per group, two‐way ANOVA. (F) Evaluation of tumor antigen loading in sEVs. RNAs from HBVc‐PEP3 overexpression cells and sEVs collected from the supernatants of indicated cell lines were analyzed by RT‐PCR to detect the levels of HBVc‐PEP3 mRNA. n=3 independent samples per group, unpaired two‐tailed t test. (G‐L) GL261‐EGFRvIII cells were implanted into C57 mice to build tumor bearing mouse model, 7 days post implantation, mice were treated with indicated reagents every three days for connective three times through i.v. injection. (G) IVIS imaging of tumor volume. Tumor volumes were monitored every five days from 7 days post tumor cells transplantation. (H) Tumor growth curve. n=5 mice per group, one‐way ANOVA. (I) Mice survival curve. n=8 mice per group, Kaplan‐Meier. (J) Histological staining analysis of tumor tissue. Scale bar=200 µm. (K) Analysis of tumor cell apoptosis after different treatments. Scale bar=100 µm. (L) Quantification of TUNEL positive cells. Four fields were randomly selected from each mouse. n=5 mice per group, one‐way ANOVA. All results are expressed as mean ± SD, a= p < 0.05, b= p < 0.01, c=p<0.001, d= p < 0.0001, ns=not significant.

Immunotherapy has been the most rapidly developing and promising cancer treatment in recent decades. Unlike traditional radiotherapy and chemotherapy, immunotherapy primarily works by modulating the immune system to attack cancer cells. cGAS/STING pathway is an attractive target for cancer immunotherapy. STING activation can not only enhance innate immune responses and reshape the TME, but also further boost anti‐tumor immune responses [35, 36, 37]. Intratumoral injection of STING agonists can effectively trigger immune responses within the tumor [38, 39, 40, 41]. However, this way of administration is technically challenging and risky for gliomas. Intravenous administration, on the other hand, may pose safety concerns [42]. Therefore, developing of method for targeted delivery of STING agonists to the tumor is of great clinical value. Endothelial cells are located at the interface between the periphery and the CNS, which endow them a vital role in the communication between peripheral immune response and neuroinflammation [20]. It has been reported that after intratumoral injection of STING agonist, endothelial cells are the main source of type I interferon secreted within the tumor [43]. Moreover, endothelial STING expression was critical for STING agonist–induced antitumor activity after intratumoral administration [44]. Our findings also confirm that endothelial cells exhibit strong expression of STING downstream molecules upon stimulation with STING agonists, which is similar to macrophages. The expression of inflammatory factors such as IFN‐β, TNF‐α, and MIP‐1α is even stronger in endothelial cells than in macrophages. Moreover, the activation of both macrophages and endothelial cells shows a clear dependence on the concentration of STING agonist (Figure S10). This indicates that a high concentration of STING agonist in the tumor is crucial for intratumoral STING activation. Through transcriptome analysis of endothelial cells, we found that the expression of numerous inflammatory and chemotactic factors in endothelial cells was significantly increased after STING activation, and the expression of cell adhesion molecules such as ICAM1 also rose significantly (Figure 5A,B). These results indicate that STING activation in VECs may activate other immune cells within the tumor by secreting large amounts of inflammatory factors. Meanwhile, STING‐activated VECs may also recruit peripheral immune cells through secreting chemokines and mediate their infiltration into the tumor through highly expressed adhesive molecules, thereby remodeling the TME and providing an ideal environment for inducing adaptive immunity. Activated antigen‐presenting cells can carry the antigens to peripheral lymphoid tissues or tertiary lymphoid tissues, where they prime tumor antigen‐specific T cells.

As we know, the existence of abundant antigens with strong immunogenicity is also important for the efficient inducing of antigen‐specific immune responses [45, 46]. For this purpose, PEP‐3, a synthetic peptide encompassing a tumor‐specific mutated segment of the epidermal growth factor receptor type vIII (EGFRvIII), was fused with hepatitis B virus core (HBVc) protein to enhance immunogenicity [47, 48]. HBVc‐PEP3 was loaded together with cGAMP, a STING agonist, into the IGFBP7‐modified sEVs, and delivered to glioma VECs through intravenous injection. Glioma VECs STING activation will reprogram TME, recruiting antigen‐presenting cells (APCs) to the tumor, which then phagocytize HBVc‐PEP3 in TME and induce strong tumor antigen specific immune response (Figure 5C). To verify this hypothesis, we prepared IGFBP7 modified sEVs loaded with HBVc‐PEP3 and STING agonist, and validated their therapeutic effects on glioma through in vitro and in vivo experiments (Figure 5D). First, bEnd.3 cells were treated with either free cGAMP or IGFBP7‐sEVs loaded with an equivalent dose of cGAMP, and the activation of cells was analyzed by RT‐PCR. The results showed that agonist‐carrying sEVs elicited endothelial activation comparable to that achieved with the corresponding dose of free agonist (Figure 5E), confirming the accuracy of our drug‐loading quantification. Numerous studies have demonstrated that endogenously overexpressed mRNAs can be packaged into exosomes, delivered to recipient cells [49], translated there into proteins, which can then function as antigens to activate immune cells [50]. We validated this conclusion using a luciferase reporter assay (Figure S11A,B). Then, HBVc‐PEP3 mRNA was packaged into sEVs by this approach, and the successful loading of HBVc‐PEP3 mRNA was verified by RT‐PCR (Figure 5F). The loading efficiency was approximately 0.05% (Figure S11C). Upon uptake of HBVc‐PEP3–loaded sEVs, macrophages became activated, indicating that the delivered HBVc‐PEP3 mRNA was successfully translated within the recipient cells (Figure S11D). These results lay a solid foundation for subsequent in vivo therapeutic studies. Next, we verified the therapeutic effect of STING agonist‐loaded sEVs on glioma through an orthotopic glioma model. Treatment with empty sEVs or sEVs carrying only HBVc‐PEP3 had little inhibition on glioma growth, indicating that remodeling of the TME is extremely important for immunotherapy. Tumor growth was markedly suppressed in mice treated with either free cGAMP or sEVs loaded with cGAMP, with sEVs loaded with cGAMP producing the more pronounced inhibition effect. Treatment with sEVs loaded with both cGAMP and tumor antigen further amplified this antitumor effect (Figure 5G,H). Both the sEVs‐cGAMP and sEVs‐cGAMP‐PEP3 ‐treated groups exhibited a significant extension in overall survival (Figure 5I). Probably the delivery of STING agonist through IGFBP7‐modified sEVs increased the concentration of STING agonist in the tumor and also enhanced the stability of the STING agonist in the body, leading to more effective activation of glioma VECs. Pathological analysis of the tumor tissues further verified the above results, with large areas of tissue damage and massive cell apoptosis appearing in the tumor of mice treated with sEVs‐cGAMP or sEVs‐cGAMP‐PEP3. Among them, sEVs‐cGAMP‐PEP3 treatment induced more tumor cell death (Figure 5J–L). These results suggest that IGFBP7‐modified sEVs efficiently delivered STING agonist and tumor antigen to the TME, generating toxicity to the tumor cells and suppressing the growth of glioma. However, further studies are needed to clarify the underlying mechanism. Throughout the entire treatment course, no significant differences in body‐weight change were observed among the various treatment groups compared with the PBS‐treated group, indicating that the different treatments did not produce notable side effects in the mice (Figure S12A). Histological results also support this conclusion, with no obvious tissue damage found in the peripheral tissues of mice with different treatments (Figure S12B).

### 2.5 Delivering of STING Agonist and Glioma Antigen by IGFBP7‐Modified sEVs Induces a Strong Tumor‐Specific Immune Response

**FIGURE 6:**
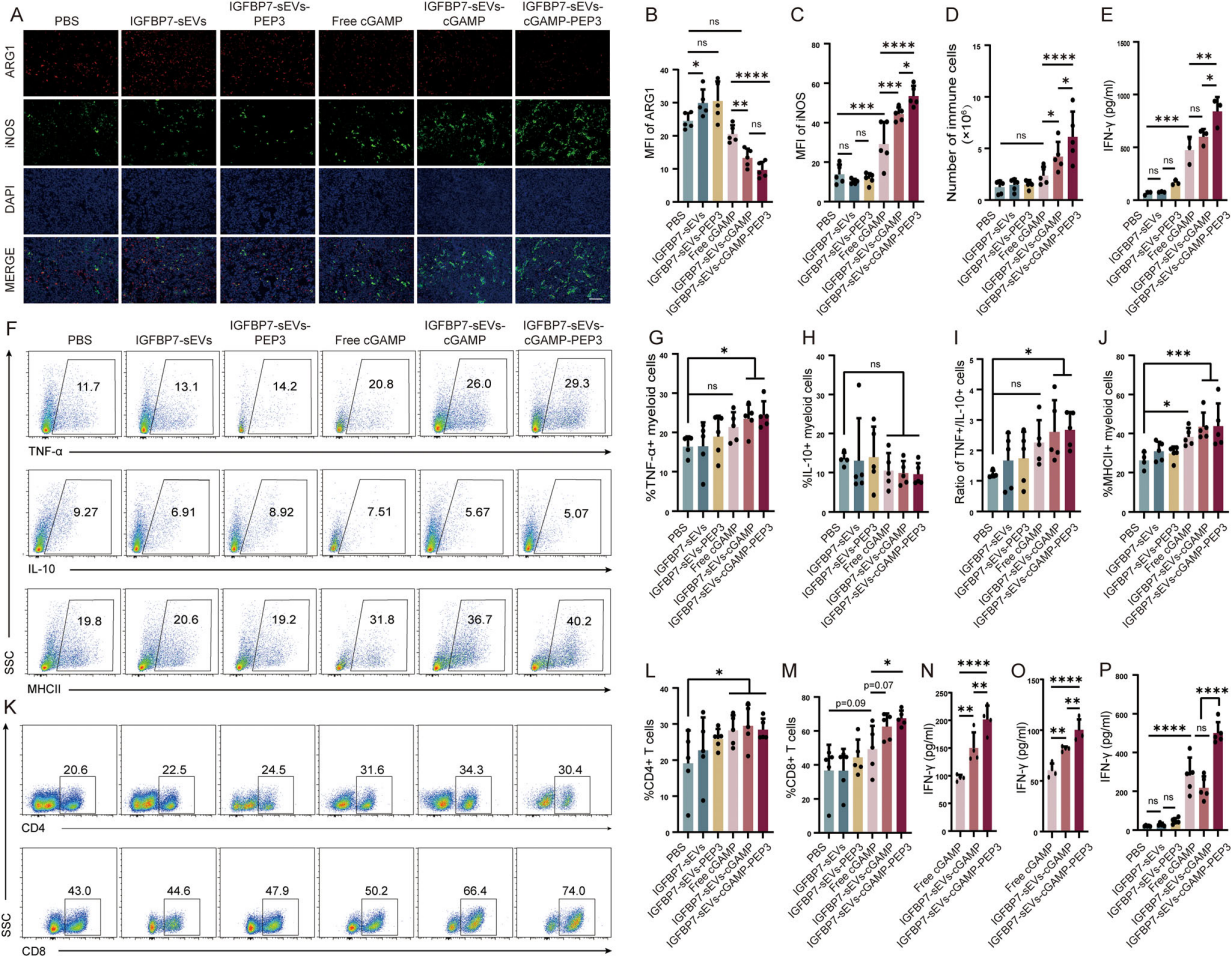
sEVs Carrying cGAMP and Tumor Antigen Induce Strong Tumor Specific Immune Response. 23 days after tumor cell transplantation, mice were sacrificed, brain, spleen and lymph node were removed and analyzed. (A) Immune fluorescence staining analysis of proinflammatory (iNOS, green) and anti‐inflammatory (ARG1, red) immune cells in the tumor. Scale bar=100 µm. (B,C) Quantitative analysis of ARG1^+^ cells (B) and iNOS^+^ cells (C). n=5 mice per group, one‐way ANOVA. (D) Number of tumor‐infiltrating immune cells. n=5 mice per group, one‐way ANOVA. (E) Elisa analysis of brain immune cells activation. Tumor infiltrated immune cells were isolated by 30/70% Percoll density gradient centrifugation. 5 × 10^5^ cells were seeded in 96‐well plate and cultured for 48 h, the concentration of IFN‐γ in each supernatant was analyzed by ELISA. n=3 mice per group, one‐way ANOVA. (F‐M) Flow cytometry analysis of tumor infiltrated myeloid cells (F) and lymphocyte (K). Immune cells isolated from the tumors were treated with PMA (50 ng/ml), ionomycin (500 ng /ml) and GolgiPlug (1 µg/ml) for 4 h, then cells were successively stained with surface and intracellular antibodies, and analyzed by flow cytometry. The percentage of TNF‐α^+^ (G) or IL‐10^+^ (H) myeloid cells were quantified, and the ratio of TNF‐α^+^ to IL‐10^+^ myeloid cells was calculated (I). (J) Quantification of MHCII positive myeloid cells. (L, M) The percentages of CD4^+^ (L) or CD8^+^ (M) T cells in lymphocytes were quantified. G‐J, L, M, n=5 mice per group, one‐way ANOVA. (N, O) Tumor antigen specific immune response assay. CD8^+^ T cells were isolated from tumor infiltrated immune cells using CD8a Microbeads, and co‐cultured with dendritic cells pre‐pulsed with PEP‐3 peptide (N) or GL261‐EGFRvIII cell line (O) for 48 h, the release of IFN‐γ was analyzed by ELISA. n=4 mice per group, one‐way ANOVA. (P) Elisa analysis of peripheral immune cells activation. Peripheral immune cells were isolated from the spleen and lymph node of mice. 5 × 10^5^ cells were seeded in 96‐well plate and cultured for 48 h, the concentration of IFN‐γ in each supernatant was analyzed by ELISA. n=5 mice per group, one‐way ANOVA. All results are expressed as mean ± SD, ^*^ *p* < 0.05, ^**^ *p* < 0.01, ^***^ *p* <0.001, ^****^ *p* < 0.0001, ns=not significant.

To figure out the mechanisms by which different treatments inhibit the growth of gliomas, we analyzed the immune cells in the tumor and in the periphery of mice from different treatment groups. First, we used iNOS and ARG1 staining to analyze the proportions of pro‐inflammatory and anti‐inflammatory immune cells in the tumor tissues. Intravenous injection of free STING agonist slightly increased the number of pro‐inflammatory immune cells and decreased the number of anti‐inflammatory immune cells (Figure 6A–C). This reflects the overall enhancing of proinflammatory activation after intravenously injection of STING agonist. However, treatment with sEVs loaded with STING agonist increased the ratio of pro‐inflammatory and anti‐inflammatory immune cells more significantly (Figure 6A–C). This result validates our above hypothesis that STING agonist delivered by modified sEVs can enhance immune activation in the tumor. Further analysis of immune cells by flow cytometry also supported this conclusion. Treatment with sEVs loaded with cGAMP significantly increased the infiltration of immune cells compared with treatment with free cGAMP (Figure 6D). Meanwhile, delivery of cGAMP by modified sEVs also slightly enhanced the secretion of IFN‐γ, while simultaneous delivery of cGAMP and glioma‐associated antigen PEP‐3 further increased IFN‐γ secretion (Figure 6E). There are more proinflammatory myeloid cells in the tumor of sEVs‐cGAMP treated mice, addition of PEP‐3 has no increase on the activity of myeloid cells (Figure 6F–I). Compared with free cGAMP treated mice, sEVs‐cGAMP or sEVs‐cGAMP‐PEP3 treatment enhanced the antigen presentation ability of myeloid cells (Figure 6F,J). Moreover, the proportion of NK cells was also slightly elevated in sEVs‐cGAMP and sEVs‐cGAMP‐PEP3‐treated groups (Figure S13). The proportion of CD4^+^ T cells within tumor tissues increased in free cGAMP, sEVs‐cGAMP, and sEVs cGAMP‐PEP3 treated groups compared with PBS treatment, but no difference was found between these three groups (Figure 6K,L). In contrast, the percentage of CD8^+^ T cells among tumor‐infiltrating lymphocytes rose modestly after sEVs‐cGAMP or sEVs‐cGAMP‐PEP3 administration (Figure 6K,M), compared with free cGAMP treatment. However, no significant change in the proportion of CD8^+^ T cells was observed between the sEVs‐cGAMP and sEVs‐cGAMP‐PEP3 treatment groups. These results indicate that targeted delivery of the STING agonist to glioma‐associated endothelial cells via engineered sEVs remodels the immune microenvironment more effectively than free STING agonist and markedly increases CD8^+^ T cell proportion. To evaluate tumor antigen‐specific CD8^+^ T cell generation, intratumoral CD8^+^ T cells were isolated and co‐cultured with either PEP‐3‐pulsed dendritic cells or GL261‐EGFRvIII cells. IFN‐γ release was measured as a read‐out of antigen‐specific reactivity. CD8^+^ T cells from sEVs‐cGAMP‐treated tumors produced more IFN‐γ upon PEP‐3 restimulation than free cGAMP‐treated group, and this response was further amplified in the sEVs‐cGAMP‐PEP3 group (Figure 6N, O), confirming an enrichment of tumor antigen specific CD8^+^ T cells. We also analyzed the activity of immune cells in peripheral immune organs. As the result shown, there was no significant difference in the activation of peripheral immune cells between free cGAMP and sEVs‐cGAMP treatment. The activity of peripheral immune cells in mice treated with sEVs‐cGAMP was even slightly lower than that in the free cGAMP‐treated group (Figure 6P). This may indicate that the STING agonist delivered by modified sEVs has a more specific immune activation in the tumor compared with free STING agonist. In summary, delivery of STING agonist through modified sEVs enhances the enrichment of STING agonist in the tumor, promotes the reshaping of TME, boosts tumor antigen presentation, increasing the activating of tumor antigen specific T cells. The addition of tumor antigen to STING agonist further enhances the generation of tumor‐specific immune response.

### 2.6 Tumor Endothelial STING Activation is Necessary for Reshaping TME

**FIGURE 7:**
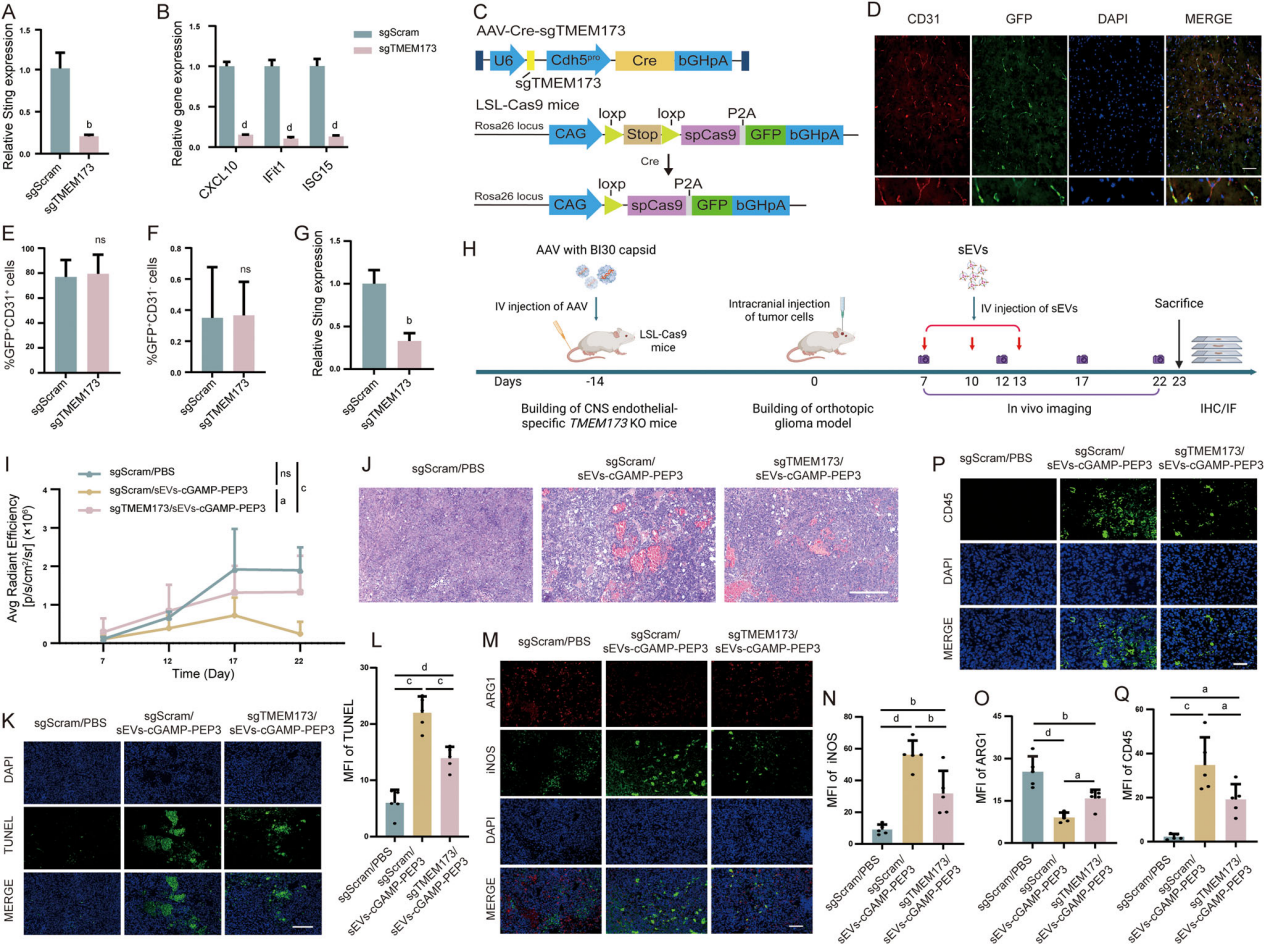
Removing of STING in CNS VECs Impairs sEVs Carrying STING Agonist and Tumor Antigen Mediated Glioma Suppression. (A) Detection of CRISPR mediated *TMEM173* knockout by RT‐PCR. n=3 independent samples per group, unpaired two‐tailed t test. (B) Effect of *TMEM173* knockout on the activation of endothelial cells by STING agonist. *TMEM173* knockout and control endothelial cells were stimulated with 10 µg/mL cGAMP for 48 h, the expression of STING downstream genes was analyzed by RT‐PCR. n=3 independent samples per group, unpaired two‐tailed t test. (C) Cartoon shows the strategy of CRISPR mediated endothelial‐specific *TMEM173* knockout in vivo. sgTMEM173 and Cre was intravenous delivered to the CNS endothelial cells of LSL‐Cas9‐GFP transgenic mice through AAV with BI30 capsid, which has been reported to have CNS endothelial cell tropism. Cas9 and GFP expression were induced by Cre whose expression was driven by Cdh5 promoter, an endothelial cell specific promoter. (D) Immunofluorescence analysis of the co‐localization of virus (GFP, green) and endothelial cells (CD31, red). Scale bar=50 µm. (E, F) The infection efficiency and specificity were quantified by calculating the percentage of GFP^+^ endothelial cells (E) and GFP^+^ non‐endothelial cells (F). n=3 mice per group, unpaired two‐tailed t test. (G) Evaluation of in vivo *TMEM173* knockout efficiency. CNS endothelial cells were isolated from brain using CD31 microbeads, and the expression of STING was analyzed by RT‐PCR. n=8 mice per group, unpaired two‐tailed t test. (H) Schematic diagram of experiment timeline. CNS endothelial cell‐specific *TMEM173* knockout mice was built by intravenous injection of AAV‐Cre‐sgTMEM173, GL261‐EGFRvIII cells were implanted into the mice to build tumor bearing mouse model two weeks post virus injection. 7 days post tumor cell implantation, mice were treated with indicated reagents every three days for three connective times through i.v. injection. (I) Tumor growth curve. n=5 mice per group, one‐way ANOVA. (J) Histological staining analysis of tumor tissue. Scale bar=200 µm. (K) Immunofluorescence staining analysis of tumor cell apoptosis after different treatments. Scale bar=50 µm. (L) Quantitative analysis of TUNEL positive cells. n=5 mice per group, one‐way ANOVA. (M) Immunofluorescence staining analysis of proinflammatory (iNOS, green) and anti‐inflammatory (ARG1, red) immune cells in the tumor. Scale bar=50 µm. (N, O) Quantitative analysis of iNOS^+^ cells (N) and ARG1^+^ cells (O). n=5 mice per group, one‐way ANOVA. (P) Immunostaining analysis of immune cell infiltration. Brain tissues were stained with CD45 antibody to analyze the intratumoral infiltration of immune cells. Scale bar=50 µm. (Q) Quantitative analysis of CD45 positive cells. n=5 mice per group, one‐way ANOVA. All results are expressed as mean ± SD, a= *p* < 0.05, b= *p* < 0.01, c= p<0.001, d= *p* < 0.0001, ns=not significant.

Although the modification of sEVs increased their uptake by glioma vascular endothelial cells, uptake by other cell types was not completely abolished. Off‐target uptake by peripheral immune cells, tumor‐infiltrating immune cells, and neoplastic cells may also enhance intratumoral inflammation. Our data reveal that 6 % of CD31^−^ cells carry PKH26 fluorescent signals, which indicates the uptake of sEVs. To verify whether the enhanced immune response of mice is mediated by endothelial STING activation, but not other intratumoral immune cells like TAMs, we examined the therapeutic effect of drug‐loaded sEVs in mice with CNS endothelial‐specific STING knockout. We drew on the previously established rapid in vivo targeted gene knockout technique to build CNS endothelial‐specific STING knockout mice [51]. First, we screened sgRNA targeted to transmembrane protein 173 (*TMEM173*), the gene encoding STING protein, and validated STING knockout in endothelial cells in vitro (Figure 7A; Figure S14). Knockout of *TMEM173* gene inhibited the activation of endothelial cells by the STING agonist (Figure 7B). Next, adeno‐associated virus (AAV) with tropism for CNS VECs [52] was used to deliver the Cre gene and *TMEM173*‐targeting sgRNA into endothelial cells, and an endothelial cell‐specific promoter Cdh5 [53] was used to drive Cre expression to further enhance the cell specificity of Cre expression. After the virus was intravenous injected into LSL‐Cas9 mice, Cre induces specific expression of Cas9 in CNS VECs, thereby achieving CNS endothelial specific knockout of STING (Figure 7C). Immunofluorescence staining results showed that the virus infected CNS VECs with high specificity and efficiency (Figure 7D–F), and the expression of *TMEM173* was blocked with high efficiency (Figure 7G). Then, glioma‐bearing mouse models were established using mice with CNS endothelial‐specific STING knockout, and treated with drug‐loaded sEVs or PBS to investigate the effects of various treatments on tumors in the context of endothelial‐specific STING deficiency (Figure 7H). The results showed that CNS endothelial‐specific knockout of *TMEM173* gene significantly attenuated the suppression of glioma growth (Figure 7I–L) and the reshaping of TME (Figure 7M–Q) by sEVs carrying STING agonist and HBVc‐PEP3. These results indicate that IGFBP7‐modified sEVs carrying STING agonist and HBVc‐PEP3 remodels the TME through activation of STING signaling in glioma endothelial cells.

### 2.7 Intravenous Delivery of STING Agonist by Modified sEVs Improves the Activity of T Cells

**FIGURE 8:**
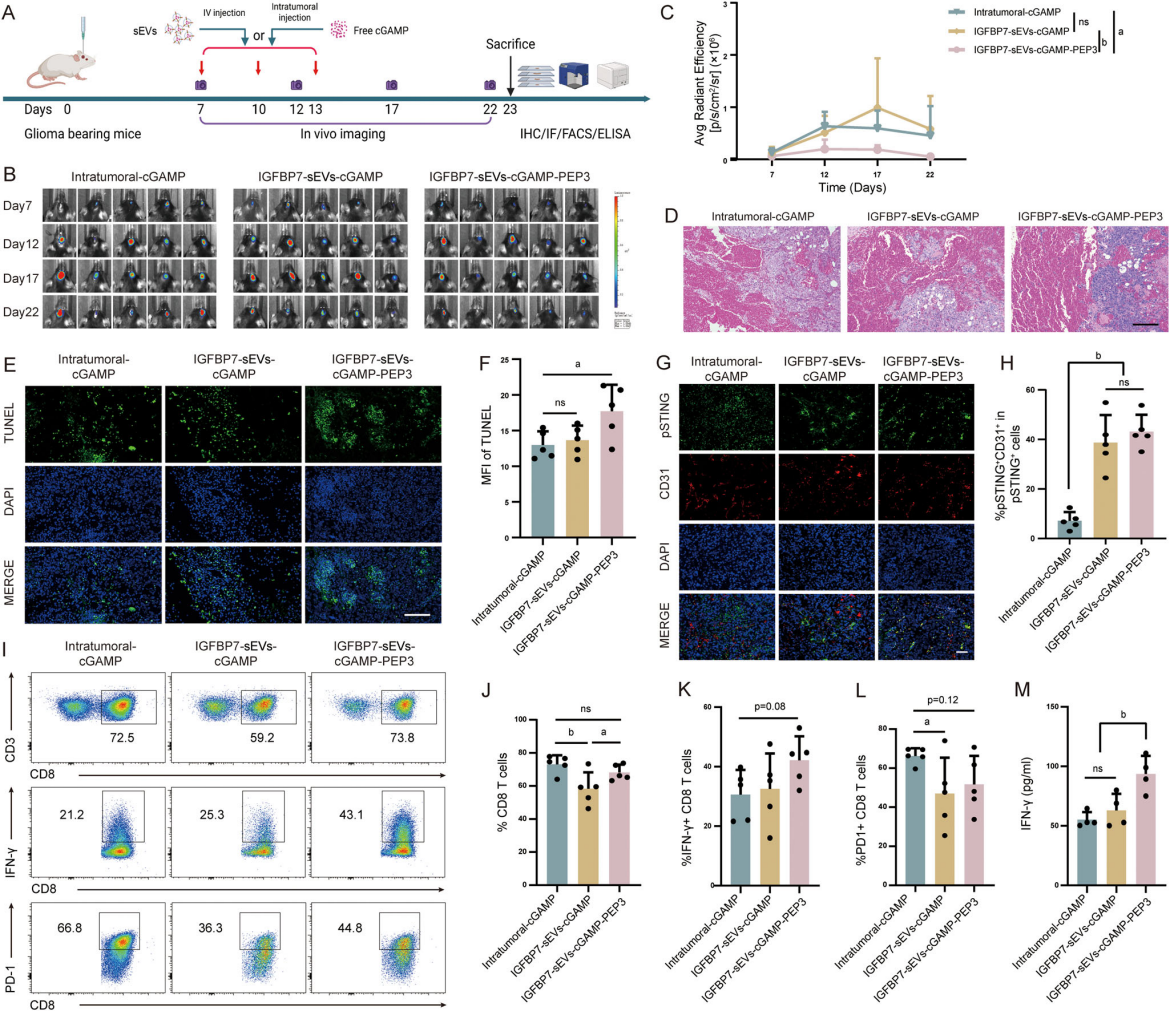
Comparison of the Therapeutic Effects Between i.v. Injected sEVs Carrying cGAMP and Intratumoral Injected Free cGAMP. (A) Schematic diagram of experiment timeline. (B) IVIS imaging evaluation of tumor volume. Tumor volume was monitored every five days from 7 days post tumor cells inoculation. (C) Tumor growth curve. n=5 mice per group, one‐way ANOVA. (D) Histological staining analysis of tumor tissue. Scale bar=100 µm. (E) Immunofluorescence staining analysis of tumor cell apoptosis after different treatments. Scale bar=50 µm. (F) Quantitative analysis of TUNEL positive cells. n=5 mice per group, one‐way ANOVA. (G) Evaluation of STING activation in the tumor. Tumor slices were co‐stained with pSTING (green) and CD31 (red) antibodies to analyze the specificity of STING activation in different groups. Scale bar=50 µm. (H) The number of pSTING‐positive cells and the number of pSTING‐positive cells among the CD31‐positive cells were quantified, and the percentage of pSTING^+^CD31^+^ cells relative to the pSTING^+^ cells was calculated. n=5 mice per group, one‐way ANOVA. (I) Flow cytometry analysis of tumor infiltrated CD8^+^ T cells. Immune cells isolated from the tumors were treated with PMA (50 ng/ml), ionomycin (500 ng /ml) and GolgiPlug (1 µg/ml) for 4 h, then cells were successively stained with surface and intracellular antibodies, and analyzed by flow cytometry. The percentage of CD8^+^ T cells in lymphocytes (J), IFN‐γ^+^ in CD8^+^ T cells (K), PD‐1^+^ in CD8^+^ T cells (L) were quantified. n=5 mice per group, one‐way ANOVA. (M) Tumor antigen specific immune response assay. CD8^+^ T cells were isolated from tumor infiltrated immune cells using CD8a Microbeads, and co‐cultured with CT2A‐EGFRvIII cell line for 48 h, the release of IFN‐γ was analyzed by ELISA. n=4 mice per group, one‐way ANOVA. All results are expressed as mean ± SD, a= *p* < 0.05, b= *p* < 0.01, ns=not significant.

Currently, STING‐targeted treatments for glioma mainly rely on intratumoral injection of STING agonists, which have shown great efficacy in inhibiting glioma growth in animal models [38, 39, 40]. However, intravenous drug administration offers significant operational advantages. How does its therapeutic effect compare with that of intratumoral injection of STING agonists? Here, we further investigated this question (Figure 8A). Meanwhile, since GL261 is a relatively immunogenic glioma model, we used the less immunogenic CT2A glioma mouse model for further confirming the therapeutic effect of glioma VECs targeted sEVs loaded with immunomodulating drugs. Compared with intratumoral injection of STING agonists, intravenous injection of sEVs carrying STING agonists produced comparable inhibitory effects on glioma growth, although intratumoral injection displayed an earlier inhibitory effect (Figure 8B,C). This might be due to the stronger innate immune response triggered by intratumoral injection of the STING agonist in the early stage. Immunohistology analysis (Figure 8D) and analysis of tumor cell apoptosis (Figure 8E,F) revealed no significant differences between the two groups. Analysis of STING activation in tumors showed that intratumoral injection of STING agonist led to more intense STING activation in the tumor, with multiple cell types exhibiting STING signal activation. In contrast, intravenous injection of sEVs carrying the STING agonist mainly activated STING signaling in glioma VECs (Figure 8G,H). Studies have reported that STING activation within CD8^+^ T cells accelerated their exhaustion [54, 55, 56, 57]. Conversely, selectively engaging STING in endothelial cells can, to some extent, reduce the activation of other cell types. We therefore compared the effects of intratumoral versus intravenous administration of a STING agonist on intratumoral CD8^+^ T cells. The results revealed that although intravenous delivery led to a modest reduction in the total fraction of tumor‐infiltrating CD8^+^ T cells (Figure 8I,J), these cells displayed a slightly higher proportion of IFN‐γ^+^ cells and a significantly lower frequency of PD‐1^+^ cells than those in the intratumoral injection group (Figure 8I,K,L). This indicates that systemic administration better preserves T‐cell activity. When the tumor antigen was co‐administered with agonist intravenously, both the abundance and activity of CD8^+^ T cells were further enhanced (Figure 8I–L). What's more, intravenously injection of agonist slightly increased tumor‐specific T cells, while intravenously co‐administration of tumor antigen with agonist generated markedly more tumor‐specific T cells (Figure 8M). Collectively, these data demonstrate that intravenous agonist delivery through sEVs can achieve therapeutic efficacy comparable to, or even superior than, direct intratumoral injection of free agonist, while more effectively safeguarding T‐cell fitness and generating tumor‐specific immunity.

## 3 Discussion

Glioma is one of the tumors with poor prognosis and high mortality in clinical practice. Due to its special spatial location, glioma is more difficult to administer drugs compared with other tumors. Intracranial drug administration has operational difficulties and risks, while intravenous drug administration is poorly absorbed due to the influence of the BBB [58]. Therefore, it is of great theoretical and practical significance to develop new targeted drug delivery methods for glioma. To overcome the barrier of the BBB to peripheral drug administration, we have chosen to target the vascular endothelial cells in the GME. This drug delivery method has two advantages. First, targeting the vascular endothelial cells in the glioma does not require crossing the BBB, which can effectively improve the efficiency of drug delivery and increase the drug concentration in the TME. Second, VECs are located at the junction between the periphery and the brain parenchyma, and play an extremely important role in the exchange of peripheral and neuro‐immune responses [16, 17, 18, 19, 20]. Targeting and intervening in the vascular endothelial cells in GME can effectively activate the immune cells in glioma and enhance the recruitment and infiltration of peripheral immune cells, reshaping TME efficiently.

During the long‐term coexistence with tumor cells, VECs are domesticated by tumor cells and develop unique transcriptional profiles [21, 22, 23]. Studies have shown that the specific high expression of CD93 in glioma vascular endothelial cells suggests that it has the potential to be a molecular target for targeting glioma vascular endothelial cells [24]. In this study, we modified sEVs with natural CD93 ligands to improve their uptake by glioma VECs with high expression of CD93. The results showed that sEVs modified with IGFBP7 have improved uptake by cells with high expression of CD93. The relatively lower uptake of MMRN2‐engineered sEVs could be attributed to decreased expression or altered trafficking of the exosomal membrane protein Lamp2b after modification. Optimizing the modification method may be able to solve this problem. However, as IGFBP7‐engineered sEVs already conferred robust uptake in vitro and in vivo, we did not seek further optimization of the MMRN2 modification strategy. sEVs surface‐modified with ligand IGFBP7 displayed robust delivery toward orthotopic gliomas after i.v. injection. Quantitative imaging revealed only minimal signals in non‐malignant brain parenchyma and the majority of peripheral tissues. Owing to the liver's filtration of venous blood, pronounced hepatic sequestration was inevitable; nevertheless, IGFBP7‐modified sEVs accumulated to a modestly lesser extent than either unmodified or MMRN2‐modified sEVs. Importantly, no significant hepatotoxicity was observed, possibly attributable to the liver's inherent immune tolerance. Collectively, IGFBP7‐modified sEVs represent an ideal vehicle for targeted drug delivery to the GME.

We next validated this platform in two clinically relevant contexts. First, we loaded a conventional chemotherapeutic drug TMZ into IGFBP7‐modified sEVs. Compared with free drug, packaging TMZ into endothelial‐targeted sEVs can not only improve the stability of TMZ in the bloodstream [59] but also markedly increase its accumulation within the tumor. It has been reported that TMZ can cross the BBB and reach tumor cell [60]. After reaching the glioma associated endothelial cells, TMZ readily diffuses across the BBB into neighboring glioma cells, thereby intensifying tumor‐cell killing at an equivalent systemic dose. Even more compelling, however, is the platform's potential for delivering immunomodulatory agents such as STING agonist. CNS VECs serve as critical coordinators of both central and peripheral immunity [16, 17, 18]; STING activated endothelial cells can express inflammatory cytokines, chemokines, and adhesion molecules. By targeting immunomodulators specifically to these cells, we can leverage the gatekeeper function of VECs to synchronize systemic and intracranial immune responses, thereby amplifying intratumoral inflammation—an effect that, to borrow a Chinese adage, “allows four ounces to move a thousand pounds.”

STING agonists have emerged as promising immunotherapeutics, with a substantial number of ongoing clinical trials administering STING agonists via direct intratumoral injection [61, 62]. For gliomas, such a delivery strategy is technically challenging and carries appreciable procedural risks. Moreover, intratumoral injection of STING agonist may introduce additional negative effects. For example, emerging evidence suggests that direct activation of STING within T cells can accelerate T‐cell exhaustion, thereby undermining the therapeutic efficacy [54, 55, 56, 57]. Recent work further indicates that, following intratumoral administration of STING agonists, endothelial cells constitute the principal source of Type I interferon production within GME [43]. Moreover, endothelial STING expression was critical for STING agonist–induced antitumor activity after intratumoral administration [44]. These observations collectively implicate tumor‐associated endothelial cells as a critical, yet underexploited, node in STING‐mediated glioma immunotherapy. In this study, we report the first time of selective STING activation in glioma associated VECs, achieved via IGFBP7 modified sEVs mediated delivery of a STING agonist. This approach circumvented the need for invasive intratumoral injection and yielded robust therapeutic benefit. Endothelial‐restricted STING signaling effectively remodeled the GME, markedly enhanced antigen‐presenting capacity of myeloid cells while simultaneously attenuated T‐cell exhaustion, subsequently amplifying tumor antigen–specific immune responses.

Nevertheless, the current work has certain limitations. In this study, we used an orthotopic syngeneic glioma model and initiated treatment on day 7 after tumor implantation. While this study can provide a preliminary evaluation of therapeutic efficacy, it has inherent limitations. Because intervention begins early, at which time tumor growth is still slow and intra‐tumoral heterogeneity is low, the strategy does not demonstrate the therapeutic response seen in highly aggressive, heterogeneous, late‐stage disease. In future studies, we will use genetically engineered orthotopic glioma models to systematically assess the therapeutic efficacy of this approach when treatment is initiated at advanced disease stages. Besides, we chose RAW264.7‐derived sEVs for the present proof‐of‐concept study because these immune cells strongly up‐regulate inflammatory cytokines upon stimulation, and the corresponding mRNAs or proteins can be packaged into secreted sEVs. Follow‐up work will specifically examine whether sEVs harvested from activated RAW264.7 cells can more effectively remodel the tumor microenvironment. It should be emphasized, however, that RAW264.7 is a mouse macrophage‐like tumor line; sEVs produced by a neoplastic cell are clearly unsuitable for clinical use. To move this technique into the clinic, appropriately selected human cell types must be employed for sEVs production. Owing to the appreciable hepatic retention of sEVs, their long‐term effects on liver homeostasis remain to be fully elucidated. Strategies such as further surface engineering of the sEVs or liver‐specific STING knockdown could mitigate this risk and will be investigated in subsequent studies. Moreover, the use of PKH26‐labeled sEVs for in vitro and in vivo investigations is inherently subject to several limitations [63]. For example, PKH26 spontaneously assembles into nano‐sized dye aggregates that are indistinguishable from labeled sEVs. These particles give false‐positive uptake/readout in both cell cultures and whole‐animal imaging. PKH26 also can rapidly associates with albumin or lipoproteins, generating fluorescent “dye‐protein complexes” that are taken up by cells and accumulate in organs, again masquerading as sEVs signals.

In summary, we have established a platform that enables highly efficient, glioma VECs‐targeted delivery of therapeutics into the GME. The approach is readily adaptable to a broad spectrum of agents and is particularly valuable for immunotherapeutic applications. Its continued refinement may offer a novel and effective strategy for treating gliomas.

## 4 Materials and Methods

### 4.1 Experimental Design

This study aimed to develop an engineered sEVs‐based targeted drug delivery system that specifically recognizes the CD93 surface marker on glioma VECs to achieve precise drug delivery to the glioma. To accomplish this goal, we employed molecular engineering techniques to fuse MMRN2/IGFBP7 with the exosomal membrane protein Lamp2b, constructing functionalized sEVs with targeting capability. These engineered sEVs were loaded with the chemotherapeutic agent TMZ or the immunomodulator STING agonist to systematically evaluate their therapeutic efficacy and safety profile. Furthermore, we established a glioma mouse model with CNS endothelial cell‐specific STING knockout, demonstrating that the immunoenhancing effects induced by modified sEVs were dependent on the activation of the endothelial STING signaling pathway, thereby mediating TME remodeling. To optimize the administration strategy, we compared intravenous versus intracranial injection of the STING agonist in the CT2A glioma model, revealing that intravenous administration more effectively induced adaptive immune responses. The therapeutic effects of the engineered sEVs were comprehensively evaluated through multiple approaches, including longitudinal tumor growth monitoring, survival analysis, ELISA quantification, and immunophenotyping analysis. The experimental design followed randomized grouping principles, with sample sizes determined based on previous literature and statistical power calculations. All experimental animals completed the predefined observational endpoints and were included in the final analysis. Investigators were blinded throughout all experiments, including treatment administration, in vivo imaging, flow cytometry analysis, and immunohistochemical evaluation. The primer sequences and related genes used in our study are provided in Tables S1 and S2. Patient sample use was approved by the Ethics Committee of Shaanxi Normal University (approval no. 2024‐104).

### 4.2 Experimental Models

#### 4.2.1 Mouse Models

C57BL/6 mice and LSL‐Cas9 mice were used in this study. C57BL/6 mice (6–8 weeks old) were purchased from the Experimental Animal Center of Shaanxi Normal University and the Experimental Animal Center of Fourth Military Medical University. LSL‐Cas9 mice were gifted from Prof. Yan Zhang, Shanghai General Hospital, China. All animal experiments were approved by the Institutional Animal Care and Use Committee (IACUC) of Shaanxi Normal University.

#### 4.2.2 Cell Lines

The cell lines, including HEK293(RRID: CVCL_0045), bEnd.3(RRID: CVCL_0170), RAW264.7(RRID: CVCL_0493), were all obtained from the American Type Culture Collection (ATCC). GL261(RRID: CVCL_Y003) and CT2A (RRID: CVCL_ZJ44) cells were gifted from Lei Zhang, Shaanxi Normal University, China. GL261 cells were genetically modified through lentiviral transduction to co‐express murine EGFRvIII and firefly luciferase genes (designated as GL261‐EGFRvIII and CT2A‐EGFRvIII, respectively). Primary CD8^+^ T cells were separated from the brain of C57BL/6J mice using the Mouse CD8 T‐Cell Isolation Kit (Miltenyi Biotec, Bergisch Gladbach, Germany). T cells were cultured in IMDM medium (Thermo Fisher Scientific, Waltham, MA, USA) supplemented with 10% FBS (Gibco, Waltham, MA, USA) and 50 µM β‐mercaptoethanol. Other cell lines were maintained in DMEM medium (Gibco, Carlsbad, CA, USA) supplemented with 10% fetal bovine serum and 1% P/S, and cultured at 37°C in a humidified 5% CO_2_ atmosphere. All cells have been tested and are free of contamination.

### 4.3 Method Details

#### 4.3.1 Plasmid Construction

The plasmid expressing Lamp2b‐RVG fusion protein was obtained from Prof. Guodong Yang, Air Force Medical University, China. The murine‐derived MMRN2 and IGFBP7 gene sequences, acquired from NCBI, were synthesized (TSINGKE Bio, Beijing, China) and subsequently ligated to the N‐terminus of Lamp2b to replace the RVG sequence, resulting in recombinant plasmids expressing MMRN2‐Lamp2b or IGFBP7‐Lamp2b fusion proteins with a C‐terminal 6×His tag. Single‐guide RNAs (sgRNA) targeting the *TMEM173* gene were designed using the Benchling bioinformatics platform, synthesized, and cloned into an AAV transfer plasmid. The kiCAP‐AAV‐BI30 plasmid was gifted from Ben Deverman (Addgene plasmid # 183749). pAdDeltaF6 was a gift from James M. Wilson (Addgene plasmid # 112867).

#### 4.3.2 Virus Preparation

The triple‐plasmid transfection system, including the transfer plasmid, the kiCAP‐AAV‐BI30 plasmid, and pAdDeltaF6, was used for AAV production. Following transfection, AAV particles were purified from the supernatant and cell lysate using iodixanol (Sigma‐Aldrich, St. Louis, MO, USA) density gradient centrifugation as previously reported [64]. Viral titers were quantitatively determined by real‐time PCR (qPCR) [64, 65].

#### 4.3.3 sEVs Preparation

First, a lentivirus carrying both the Lamp2b‐IGFBP7 and HBVc‐PEP3 expression cassettes was produced and transduced into RAW264.7 cells to build a Lamp2b‐IGFBP7 and HBVc‐PEP3 high‐expression cell line. Cells were selected with 3 µg/ml puromycin for two weeks. The supernatant was then collected and sequentially centrifuged at 300 × g for 10 min, 2000 × g for 20 min, and 10,000 × g for 30 min at 4°C to remove cellular debris. This was followed by ultracentrifugation at 100,000 × g for 70 min at 4°C to collect IGFBP7‐modified sEVs carrying HBVc‐PEP3. The pellet was washed once with PBS and subjected to another ultracentrifugation step at 100,000 × g for 70 min at 4°C. The final pellet was resuspended in PBS and stored at −20°C until further use.

#### 4.3.4 BCA Quantification of sEVs

sEVs were resuspended in 100 µL RIPA lysis buffer (Mishu Bio, Shanghai, China) supplemented with 1% protease inhibitor (Invitrogen, Carlsbad, CA, USA). The mixture was incubated on ice for 40 min, followed by centrifugation at 13,300 rpm for 10 min at 4°C to remove cell debris. The concentration of sEVs protein was then determined using a BCA protein assay kit (Mishu Bio, Shanghai, China).

#### 4.3.5 sEVs Electron Microscopy Imaging

sEVs purified from macrophage‐conditioned medium were resuspended in PBS and fixed with 2% paraformaldehyde (PFA) for 30 min at room temperature. Following fixation, the sEVs suspension was applied to TEM grids and allowed to dry for 30 min. Samples were then negatively stained with uranyl acetate. sEVs morphology was examined using an HT7700 transmission electron microscope (Hitachi, Japan) operating at an acceleration voltage of 120 kV.

#### 4.3.6 Nanoparticle Tracking Analysis

Nanoparticle tracking analysis (NTA) measurements of purified sEVs were performed using a nanoparticle tracking analyzer (Particle Metrix GmbH, Meerbusch, Germany). The ZetaView software was used to collect and analyze the concentration and size distribution of sEVs particles. All procedures were conducted at room temperature.

#### 4.3.7 Western Blot

Total protein concentration was determined using a BCA assay kit. Equal amounts of total protein from cell lysates were subjected to sodium dodecyl sulfate‒polyacrylamide gel electrophoresis (SDS‒PAGE) and transferred to a polyvinylidene fluoride (PVDF) membrane. After blocking with 5% skim milk, the membranes were sequentially incubated with primary antibodies (Table S3) and HRP‐conjugated secondary antibodies, and then visualized using ECL reagent (Mishu Bio, Shanghai, China).

#### 4.3.8 sEVs Uptake Assay

To evaluate the uptake efficiency of differentially modified sEVs by CD93‐high‐expressing cells, we established CD93‐overexpressing HEK293 cells. To minimize false‐positive signals arising from sEVs merely adsorbed to the plasma membrane, PKH26‐labeled sEVs were first incubated with CD93‐HEK293 cells for 24 h. Then cells were changed with fresh medium and incubated for an additional 2 h, followed by washing for three times with PBS before fixation and imaging.

#### 4.3.9 TMZ Loading

TMZ was loaded into sEVs as previously reported [66]. Briefly, sEVs (500 µg/mL) were co‐incubated with 500 µg temozolomide (TMZ, HY‐17364) in a total volume of 500 µL at 37°C for 30 min. Ultrasonication was performed at 25 W using a program of 30 sec on /150 sec off for 6 cycles while cooling on ice. Following sonication, sEVs were further incubated for 1 h at 37°C to facilitate membrane recovery. To remove unencapsulated TMZ, the sEVs suspension was washed by resuspending and ultracentrifuging at 120,000 × g for 70 min at 4°C in PBS. The drug loading efficiency was quantified by HPLC with ultraviolet detection at 330 nm.

#### 4.3.10 STING Agonist Loading

sEVs were loaded with STING agonists (CdGMP, HY‐107780 or cGAMP, HY‐12512) using an electroporation system [67]. Briefly, a 100 µL electroporation mixture containing sEVs (100 µg/mL) and STING agonists (100 µg/mL) was prepared and transferred into a 4 mm pre‐chilled electroporation cuvette, followed by incubation at 37°C for 30 min. Electroporation was performed at 200 V, after which the sample was incubated at 37°C for 1 h to allow membrane recovery. To remove unencapsulated agonists, the mixture was subjected to ultracentrifugation at 100,000 × g for 2 h at 4°C, washed with PBS, and resuspended to obtain purified sEVs‐cGAMP complexes. The loading efficiency of STING agonists was quantified by measuring the optical density (OD) at 245 nm.

#### 4.3.11 HPLC Analysis

HPLC analysis was performed as previously reported [68]. The mobile phase consisted of methanol and 0.5% acetic acid (30:70, v/v). The flow rate was maintained at 1.1 mL/min, with an injection volume of 20 µL. The column temperature was controlled at 35 ± 1°C. The retention time for TMZ was 3.56 min, and the total analytical run time was 8 min. Detection was performed at a UV wavelength of 330 nm. TMZ standards were prepared by diluting TMZ in PBS to concentrations of 100, 50, 25, 12.5, 6.25, and 0 µg/mL, and a calibration curve was constructed. The drug loading efficiency was calculated based on the measured sample concentrations.

#### 4.3.12 CCK‐8 Assay

GL261 cells were trypsinized and seeded into a 96‐well plate at a density of 3000 cells per well. The cells were treated with specific reagents with the concentration indicated in the figure legends and cultured for 48 h. Subsequently, 10% CCK‐8 reagent (Beyotime Bio, Jiangsu, China) was added to each well, and the plate was incubated at 37°C in a cell culture incubator for 2 h. The absorbance at 450 nm was measured using a microplate reader to calculate cell viability.

#### 4.3.13 Immunostaining Analysis

Immunofluorescence staining: Tissues embedded in OCT compound were cryosectioned (6–8 µm), fixed with 4% paraformaldehyde for 15 min, permeabilized with 0.3% Triton X‐100 for 10 min, and blocked with 5% horse serum plus 1% BSA for 30 min. Primary antibodies (Table S3) were incubated at 4°C overnight, followed by PBS washes and incubation with fluorophore‐conjugated secondary antibodies at room temperature (protected from light) for 1 h. Finally, sections were mounted with antifade mounting medium containing DAPI (Abcam, Waltham, MA, USA) and observed under a fluorescence microscope. HE staining: Tissue samples were fixed in 4% neutral buffered formalin, dehydrated through an ethanol gradient, and embedded in paraffin. Sections were then dewaxed, stained with hematoxylin (5–10 min), differentiated in 1% acid alcohol, blued in running water, and counterstained with eosin (1–3 min). After sequential dehydration and clearing, slides were mounted with neutral balsam and examined under a microscope.

#### 4.3.14 Isolation of Immune Cells from Tumor and Peripheral Immune Organs

The tumor tissue was minced and placed in serum‐free DMEM medium supplied with 0.66 mg/mL collagenase D and 8 U/mL DNase I (both from Roche, Basel, Switzerland) at 37°C for 30 min. The resulting suspension was filtered through a 70 µm nylon filter (Corning, NY, USA) to obtain a single‐cell suspension. Immune cells were separated by gradient centrifugation using 30% and 70% Percoll (GE Healthcare, 17‐0891‐02, DE, USA) gradients, and collected from the 30%–70% interphase. To isolate immune cells from peripheral immune organs, spleen and lymph node from tumor‐bearing mice were removed and gently pushed through a 40 µm cell strainer; erythrocytes were subsequently depleted with RBC Lysis Buffer (BioLegend, Dedham, MA, USA).

#### 4.3.15 Tumor Antigen Specific Immune Response Assay

Bone marrow‐derived mononuclear cells were isolated from C57BL/6J wild‐type mice and differentiated into dendritic cells (DCs) in vitro by stimulating with 20 ng/mL murine GM‐CSF and 10 ng/mL murine IL‐4. These cells were further matured by treatment with LPS (100 ng/mL for 24 h; 1 × 10^5^ cells per well). Subsequently, the mature DCs were pulsed with 100 µg/mL PEP‐3 peptide overnight and then co‐cultured with CD8^+^ T cells (2 × 10^5^ cells per well) isolated from tumor‐infiltrating immune cells using the CD8a^+^ T cell Isolation Kit (Miltenyi Biotec) for 48 h. Alternatively, a direct co‐culture experiment of CD8^+^ T cells with GL261‐EGFRvIII tumor cells (2 × 10^4^ cells per well) was conducted. All cell co‐culture experiments were performed in round‐bottom 96‐well plates (Corning, New York, USA). The supernatants from all cultures were collected, and the secretion of IFN‐γ was analyzed using a commercial mouse IFN‐γ ELISA kit (R&D Systems, Minneapolis, MN, USA).

#### 4.3.16 Flow Cytometry

For flow cytometry analysis, immune cells isolated from tumor were quantified via a hemocytometer, and 1 × 10^6^ cells from each mouse were treated with phorbol 12‐myristate‐13‐acetate (PMA; 50 ng/ml; Sigma), Ionomycin (500 ng/ml; Sigma) and BD GolgiPlug (1ug/ml; BD Bioscience) for 4 h. Cells were washed twice with 1 mL of FACS buffer (5% BSA in PBS), suspended in 100 µl of FACS buffer with surface antibodies (Table S3) and incubated at 4°C for 20 min. Then, cells were fixed in fixation buffer (Medium A) (Thermo Scientific) and stained with intracellular antibodies diluted in permeabilization buffer (Medium B) (Thermo Scientific) at room temperature, or 20 min. All samples were collected on a CytoFLEXS flow cytometer and analyzed using FlowJo software.

### 4.4 In Vivo Experiments

#### 4.4.1 Tumor‐Bearing Model

GL261‐EGFRvIII and CT2A‐EGFRvIII cells were trypsinized and resuspended in PBS at a concentration of 2 × 10^7^ cells/mL. C57BL/6J mice or AAV‐sgTMEM173‐Cre injected LSL‐Cas9 mice (8–10 weeks old) received a stereotactic intracranial injection of 5 µL (containing 1 × 10^5^ cells) cell suspension in the right striatum (2 mm lateral, 1 mm anterior to Bregma, and 3 mm deep) using a stereotaxic apparatus. Tumor growth was monitored by in vivo imaging on day 7 post‐implantation, after which the mice were randomly allocated into different groups for further studies.

#### 4.4.2 Building of CNS Endothelial‐Specific TMEM173 Knockout Mice

LSL‐cas9 mice (6–8 weeks old) were intravenously injected with AAV‐sgTMEM173‐Cre or AAV‐Scramble‐sgRNA‐Cre (1 × 10^12^ vg per mouse). 14 days post virus injection, CNS endothelial cells were isolated using CD31 microbeads (Miltenyi Biotec) and analyzed by RT‐PCR to verify TMEM173 knockout efficiency. The endothelial‐specific TMEM173 knockout mice were randomly grouped for subsequent experiments.

#### 4.4.3 Mice Treatment

Mice received PBS, free TMZ/free cGAMP, and sEVs formulations via tail vein injection: TMZ‐loaded sEVs on days 7/9/11 (at a dose of 1 mg/kg), STING agonist‐loaded sEVs on days 7/10/13 (at a dose of 3 mg/kg). For intratumoral administration of cGAMP, 15 µg cGAMP was injected into the tumor using a stereotaxic apparatus. Body weights were recorded daily, and survival was monitored for 60 connective days.

#### 4.4.4 In Vivo Imaging

The tumor growth was monitored using bioluminescence imaging. Mice bearing GL261, GL261‐EGFRvIII, or CT2A‐EGFRvIII tumors were anesthetized with isoflurane and intraperitoneally injected with D‐luciferin (150 mg/kg) 10 min before imaging. Tumor progression was monitored using the IVIS imaging system (Caliper Life Sciences, Waltham, MA, USA). The acquired data were analyzed using Living Image software.

### 4.5 Bioinformatics Analyses

#### 4.5.1 Analysis of scRNA‐Seq Datasets

The gene expression profiling interactive analysis (GEPIA) database based on TCGA was used to explore the expression of the CD93 gene in LGG and HGG. To analyze the expression of CD93 in different types of cells in glioma, single‐cell transcriptome data of 4 cases of HGG from GEO databases (GSE242044 dataset [69]) were obtained, and data analysis was performed using the Seurat 5.2.1 pipeline. First, quality control (removing cells with mitochondrial genes >10% or gene number <200) and normalization were carried out. Then, batch effects were corrected using the Harmony algorithm, doublets were removed by DoubletFinder, and UMAP dimension reduction and cell clustering (resolution = 1.2) were performed based on the top 30 Harmony dimensions. Cell clusters were annotated as endothelial (PECAM1, CD34, FLT1, KDR, VWF, CABP5, ATRX), mural cell (RGS5, HIGD1B, NOTCH3, PDGFRB, FILIP1L), meningeal fibroblast (SLP1, KCNMA1, SLC26A2, CRABP1, CHODL), macrophages (TYROBP, IL1B, AIF1, CD68, CD163), oligodendrocytes (PLP1, MBP, TF, QDPR, GNP), tumor cell (COL1A1, COL3A1, POSTN, COL6A3, COL5A1), T cell (GNLY, NKG7, GZMB, GZMA, CD3D). The expression of CD93 was then compared across these annotated cell populations.

#### 4.5.2 Transcriptomics Analysis for Endothelial Cells

bEnd.3 cells under optimal growth conditions were systematically divided into two experimental groups: (1) the treatment group exposed to STING agonist (20 µg/mL) and (2) the untreated control group. After 48 h of co‐culture, cellular lysates were prepared using TRIzol reagent (Mishu Bio, Shanghai, China) for RNA extraction. Subsequently, purified mRNA samples were subjected to transcriptome sequencing. The sequencing data were computationally analyzed through pathway enrichment analysis using KEGG (Kyoto Encyclopedia of Genes and Genomes) databases.

#### 4.5.3 Statistical Analysis

All data presented in the figures are expressed as mean ± SD. A p‐value < 0.05 was considered statistically significant. Sample size for each experiment is indicated in the figure legend. Comparisons between two groups were performed using unpaired two‐tailed Student's t‐tests, while comparisons among more than two groups were analyzed by one‐way ANOVA with Tukey's multiple comparison test or two‐way ANOVA. Graph generation was performed using GraphPad Prism 8 software. Animal survival data are presented as Kaplan‐Meier survival curves and were statistically analyzed using the log‐rank test. For quantitative analysis of the immunofluorescence results, we followed the workflow detailed below: For the control group, 7–9 fields of view per brain were acquired by systematic random sampling across the entire brain section. For tumor‐bearing animals, 7–9 fields were similarly sampled exclusively within the tumor region. For human clinical specimens, peritumoral (adjacent non‐tumor) tissues were used as the control. The image‐acquisition protocol was the same as that used for the mouse samples. All images were analyzed with ImageJ; mean fluorescence intensity was measured after automatic thresholding and background subtraction.

## Author Contributions

**D.X**., **W.Z**., **H.X**., and **H.G**. conceived and designed the experiments, analyzed data, and wrote the manuscript. **L.L**., **F.X**., **Z.Z**., **X.Y**., **F.Y**., and **P.L**. carried out the experiments. **F.X**. and **X.Y**. helped with the experimental design and statistical analysis. **Y.C**. performed flow cytometry experiments. **J.Z**., **P.Y**., **X.Z**., and **X.S**. helped in cell culture and virus preparation. **P.M**. helped with the mouse glioma model and revised the manuscript. **Q.M**., **Z.Z**., and **Y.Y**. helped with evaluating immunohistological results and revised the manuscript. **D.X**., **W.Z**., **H.X**., and **H.G**. co‐supervised the study. All authors read and approved the final manuscript.

## Funding

This work was supported by the National Natural Science Foundation of China (Nos. 82471828, 82101443), the Natural Science Basic Research Program of Shaanxi Province (2023‐JC‐YB‐160), the Young Physician Training Project of Xijing Hospital (XJZT25QN44), the Key R&D Program of Shaanxi Province (2024SF‐ZDCYL‐04‐10), Shaanxi Province Health Science Research and Innovation Capacity Enhancement Project (2025PT‐03).

## Ethics Approval

Patient sample use was approved by the Ethics Committee of Shaanxi Normal University (approval no. 2024‐104).

## Conflicts of Interest

The authors declare no conflict of interest.

## Permission to Reproduce Material from Other Sources

No copyrighted figures or materials from other sources were reproduced in this manuscript.

## Supporting information




**Supporting file**: advs73985‐sup‐0001‐SuppMat.docx.

## Data Availability

The data that support the findings of this study are available from the corresponding author upon reasonable request.
