# Supplementary material for: Engineered Small Extracellular Vesicles Targeting Tumor‐Associated Endothelial Cells to Effectively Remodel the Glioma Microenvironment
Source: Adv Sci (Weinh). 2026 Jan 21;13(18):e18490. doi: 10.1002/advs.202518490 (PMC13042902; doi:10.1002/advs.202518490)
Supplement: Supplementary file 1 — Supporting file: advs73985‐sup‐0001‐SuppMat.docx. [file ADVS-13-e18490-s001.docx]

Supporting Information

**Engineered Small Extracellular Vesicles Targeting Tumor-Associated Endothelial Cells to Effectively Remodel the Glioma Microenvironment**

*Lingling Liu^1,2†^, Feiyang Xu^2†^, Zhiming Zheng^3†^, Xiaodan Yang^2^, Fang Yang^2^, Pei Liu^2^, Yuankun Chen^2^, Yunshu Yang^1^, Junli Zhao^2^, Peiyan yang^2^, Xiaojing Zheng^2^, Xiaohong Sun^2^, Ping Mao^4^, Qinwen Mao^5^, Hao Guan^1*^, Haibin Xia^2*^, Weifeng Zhang^2*^, Dan Xiao^1,6*^*

^†^These authors contributed equally to this work.

* Corresponding authors.

**Figure S1.** Immunostaining evaluates the expression of CD93 expression in endothelial cells. Tumor slice and non-tumor slice were stained with CD93 and CD31 antibodies to analyze the expression of CD93 in tumoral VECs and normal CNS VECs. (A) Immunofluorescence staining analysis of mouse glioma tissue (NB, normal brain). Scale bar=50 μm. (B) Statistic analysis of A, n=5 mice per group. Unpaired two-tailed t-test. All results are expressed as means ± SD, ***p < 0.001.


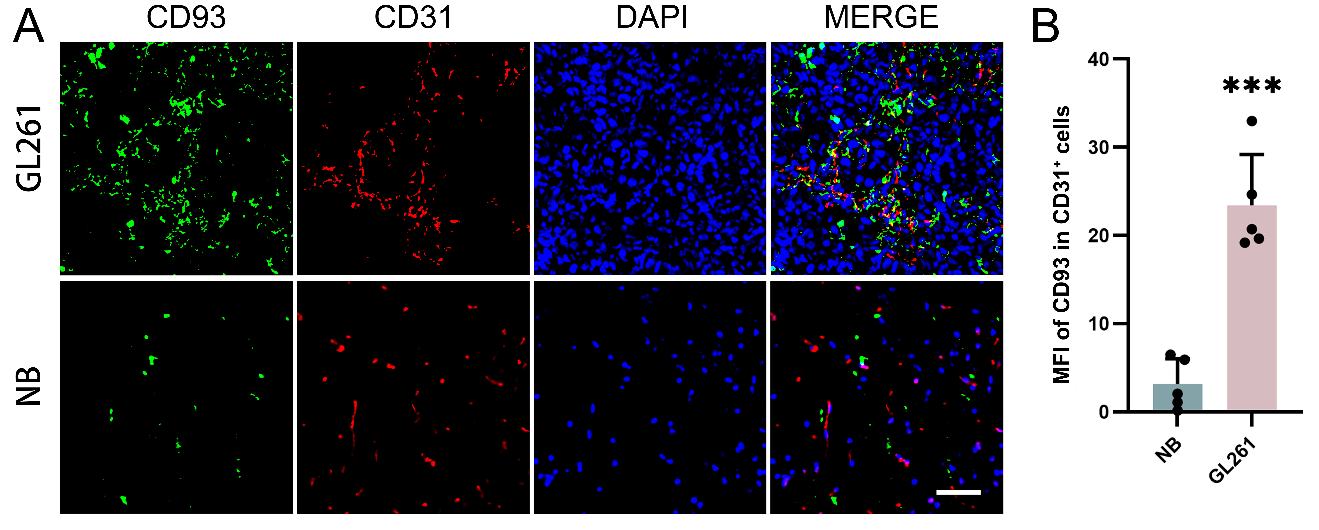


**Figure S2.** Immunostaining analysis of CD93 expression in peripheral tissues. Peripheral tissues from IGFBP7 modified sEVs injected mice were removed and stained with CD93 antibody to verify the expression of CD93 in peripheral tissues. Scale bar=100 μm.


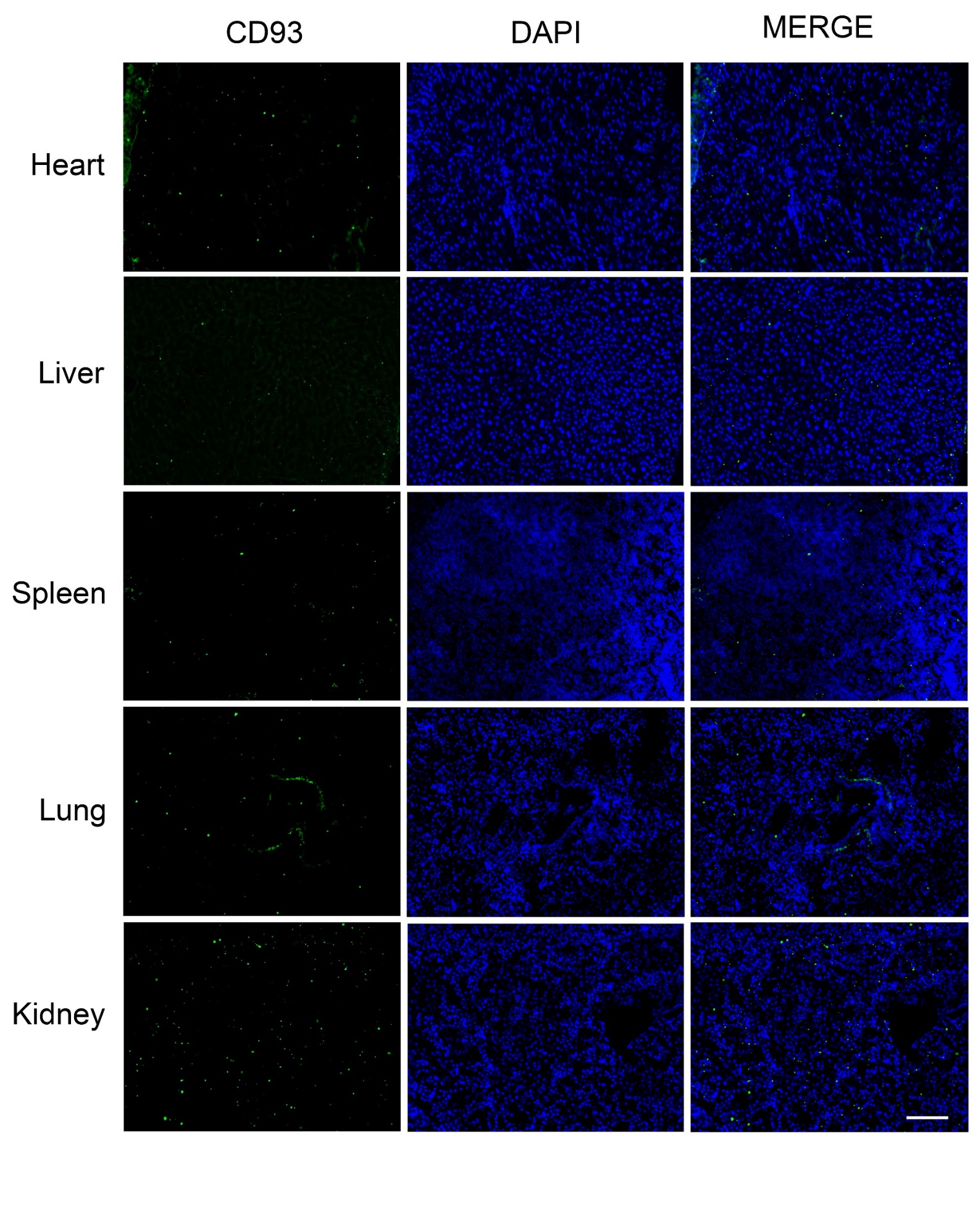


**Figure S3.** Characterization of sEVs. (A) Wide-field TEM images. Scale bar=500 nm. (B-E) uncropped western blot images.

**
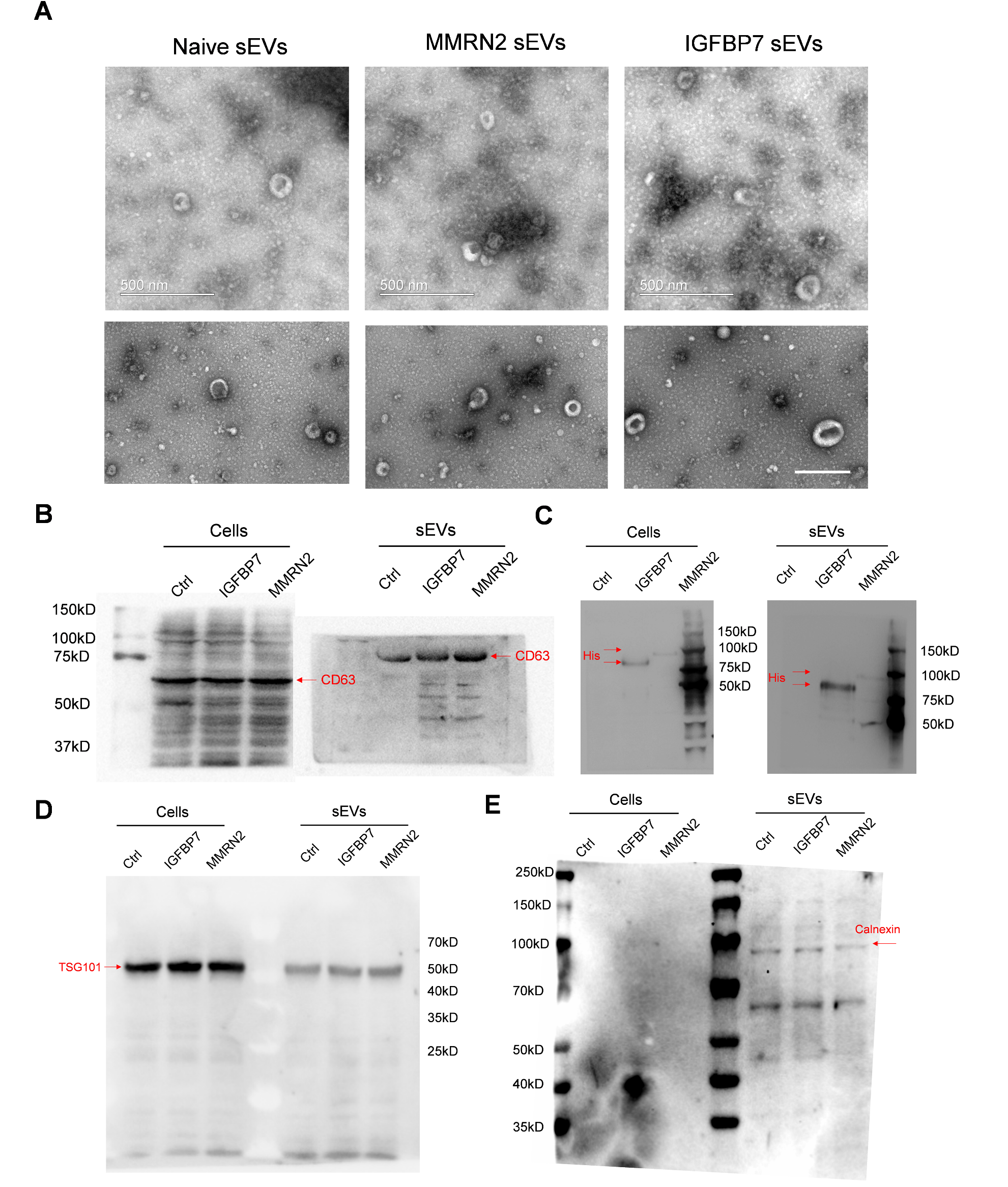
**

**Figure S4.** Evaluation of IGFBP7-lamp2b and MMRN2-lamp2b expression after transfection. (A) IGFBP7-lamp2b or MMRN2-lamp2b plasmid was co-transfected with GFP expression plasmid to verify the transfection efficiency of both plasmids. Scale bar=150 μm. (B) The expression levels of IGFBP7-lamp2b and MMRN2-lamp2b plasmid after transfection. RNA was extracted from IGFBP7-lamp2b or MMRN2-lamp2b plasmid transfected cells and the expression of lamp2b was analyzed by RT-PCR. n=3 independent samples per group, unpaired two-tailed t test. All results are expressed as means ± SD, ** p < 0.01.


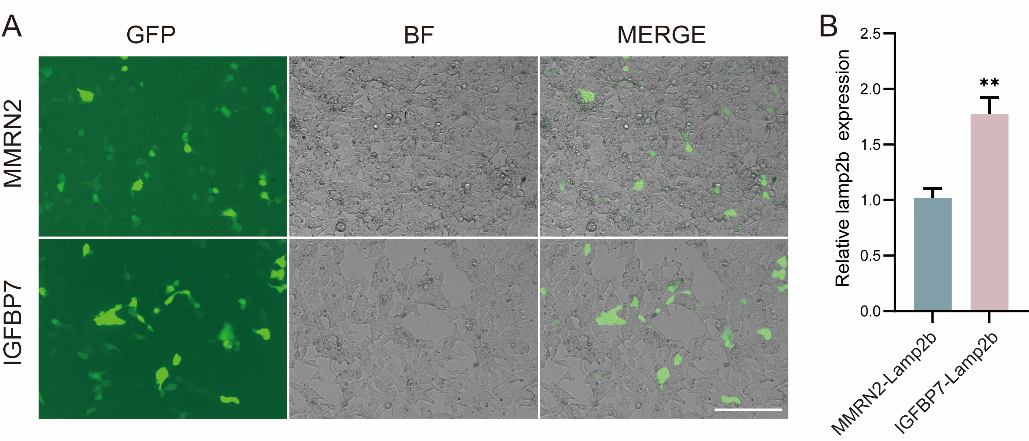


**Figure S5.** sEVs uptake efficiency in HEK293 cells. Indicated sEVs were labeled with PKH26 dye and incubated with HEK293 cells for 24 hours, then cells were washed with PBS for three times and observed under fluorescence microscope. Scale bar=50 μm.


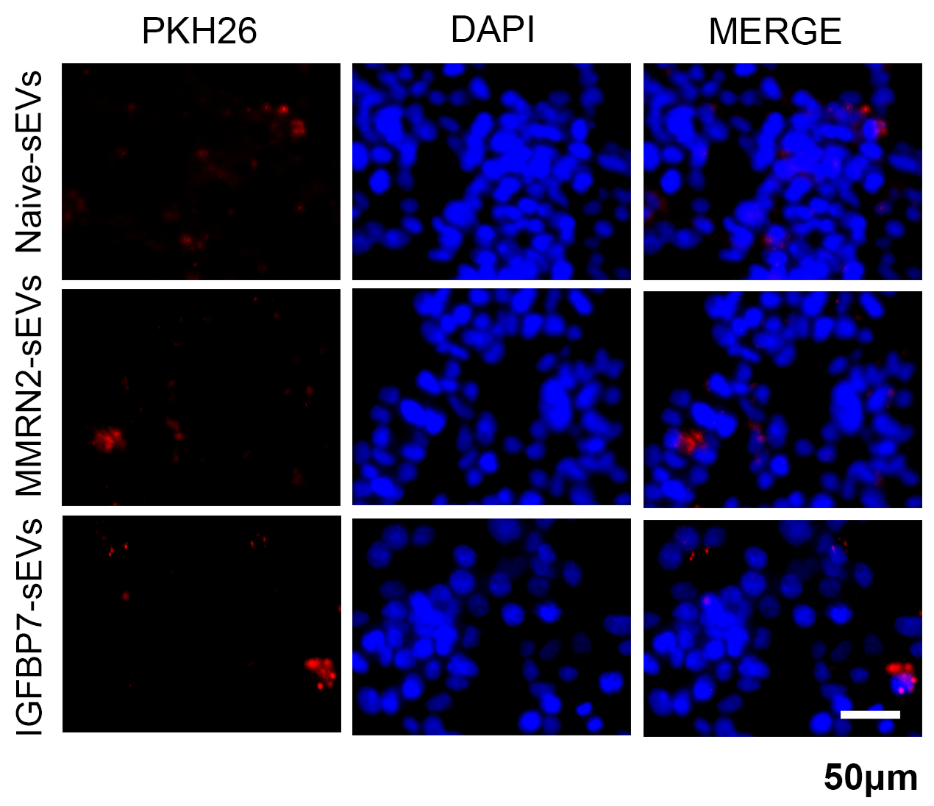


**Figure S6.** The percentage of fluorescent signal in each organ. N=3 mice per group, one way ANOVA. All results are expressed as means ± SD, * p < 0.05, ns=not significant.

**
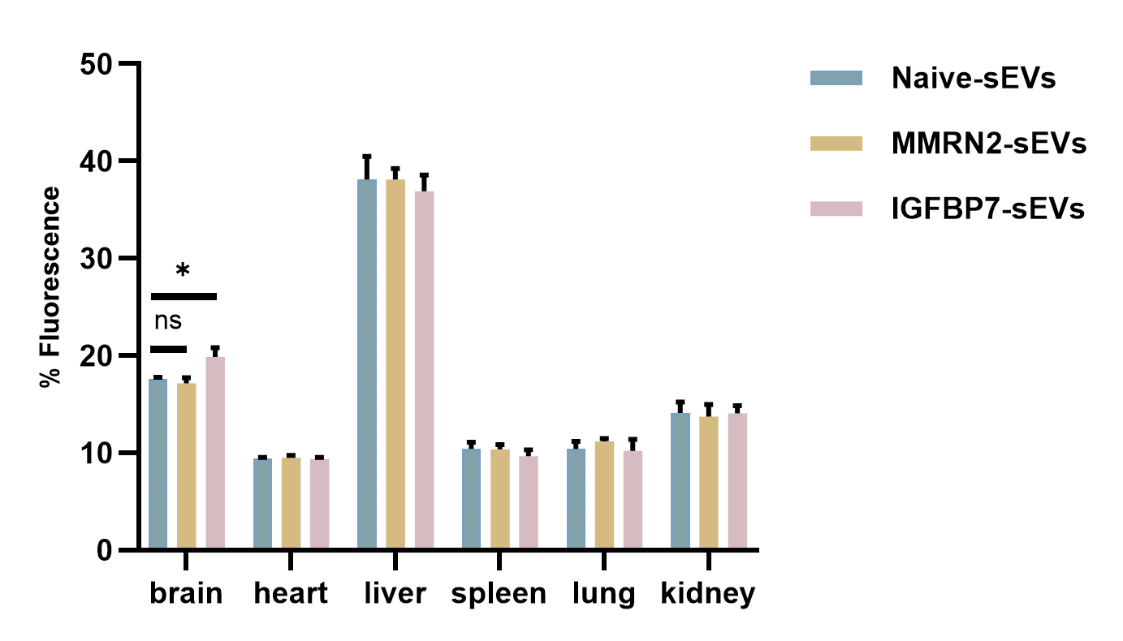
**

**Figure S7.** Analysis of the distribution of sEVs in vivo. (A) Immunostaining analysis of PKH26 in lung, heart, kidney, spleen. Indicated organs from IGFBP7-modified sEVs injected mice were removed and PKH26 fluorescence signals were detected under microscope. Scale bar=50 μm. (B) Detection of PKH26 in liver. Livers from specific sEVs injected mice were removed and stained with Iba1 to verify the localization of PKH26 in the liver. Scale bar=50 μm. (C) Quantification of the MFI of PKH26 in different tissues. (D) Quantification of PKH26 MFI in the livers of mice injected with different sEVs. n=5 mice per group. One way ANOVA. All results are expressed as means ± SD, * p < 0.05, ns=not significant.


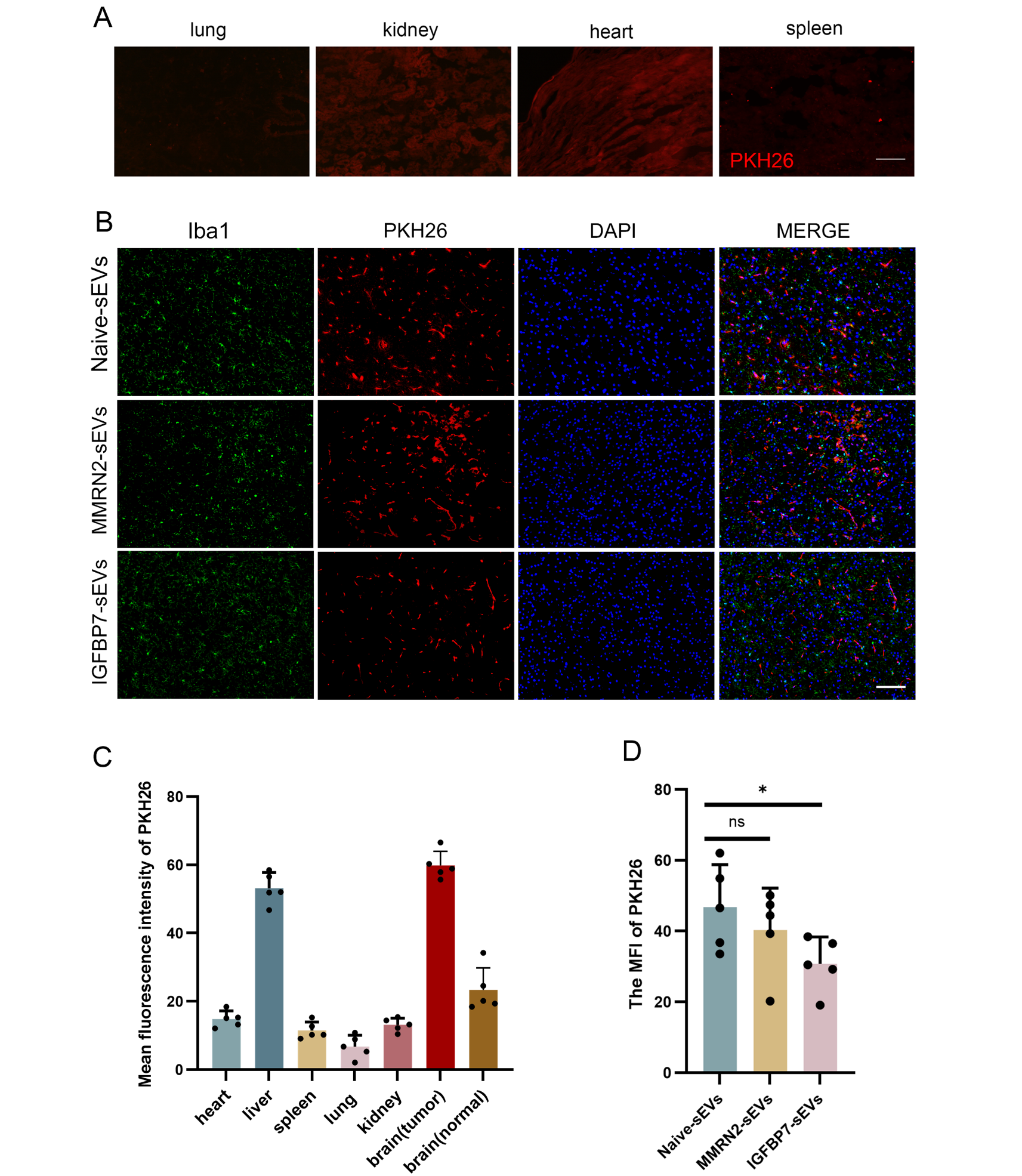


**Figure S8.** Quantification of the percentage of PKH26 positive intratumoral CD31^-^ cells. Different sEVs injected GL261 bearing mice were sacrificed and brains were removed and the percentage of PKH26^+^ cells in CD31^-^ cells were quantified. n=5 mice per group, one way ANOVA. All results are expressed as means ± SD.

**
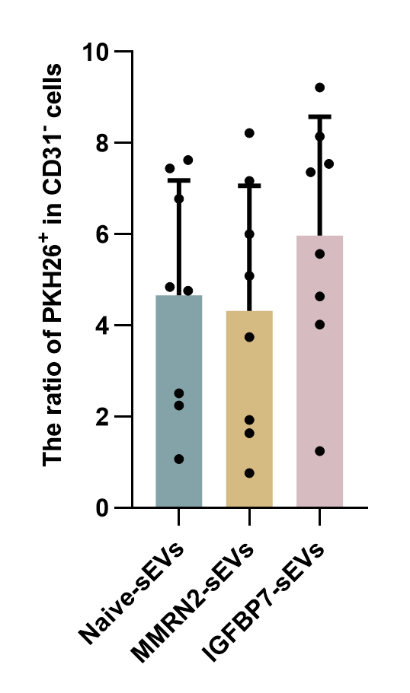
**

**Figure S9.** HE staining to evaluate the damage of peripheral tissues after treatment with different reagents. Peripheral tissues from different reagents injected mice were removed and HE staining was performed to evaluate the toxicity of different reagents to the peripheral tissues. Scale bar=100 μm.


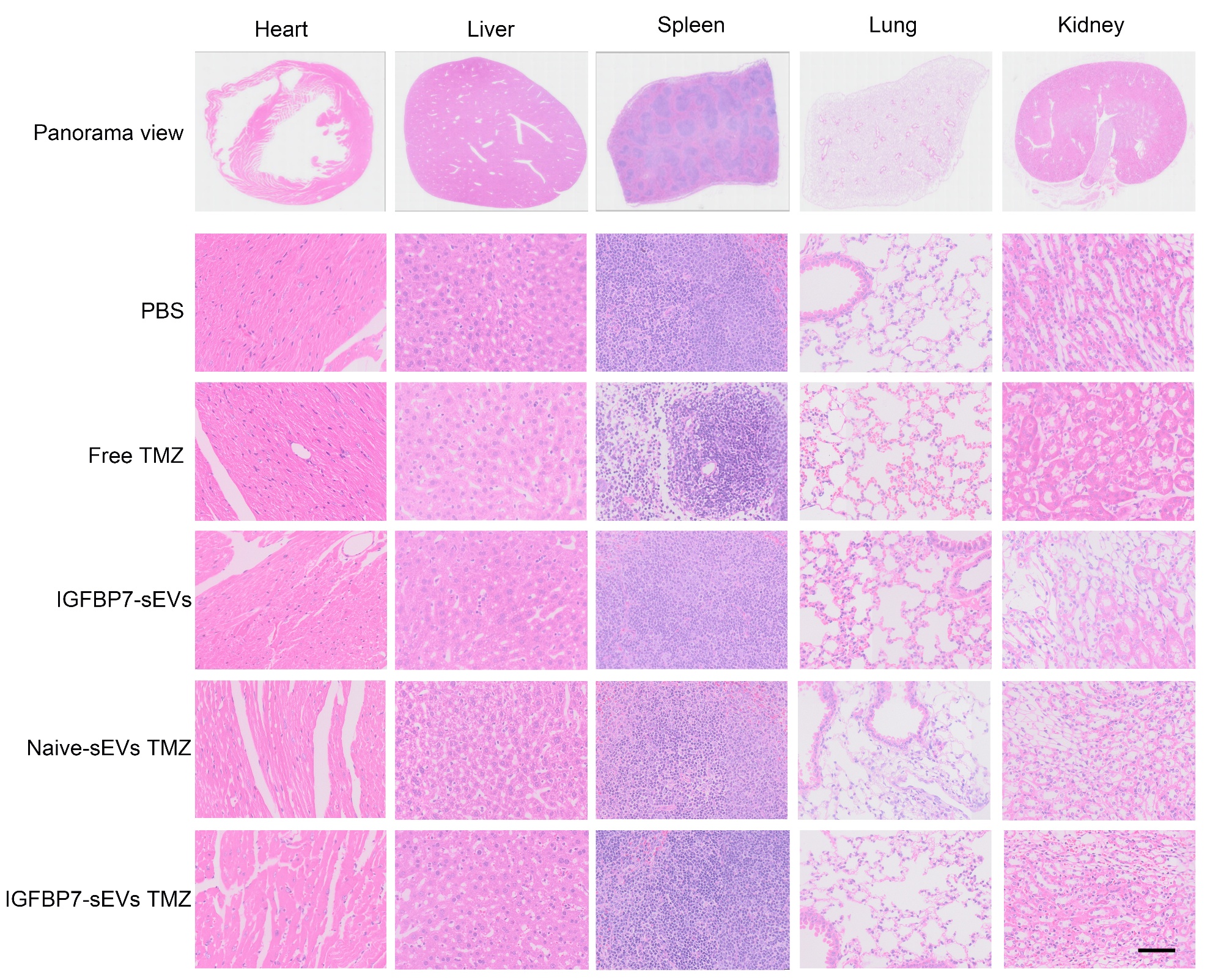


**Figure S10.** Effect of STING activation in macrophage and endothelial cells. RAE264.7 (A) and bEnd.3 (B) cells seeded in 24-well plate were stimulated with indicated concentration of cGAMP for 48 hours, the expression of STING downstream genes was analyzed by RT-PCR. n=3 independent samples per group, unpaired two-tailed t test. All results are expressed as means ± SD, * p < 0.05, **** p < 0.0001, ns= not significant.


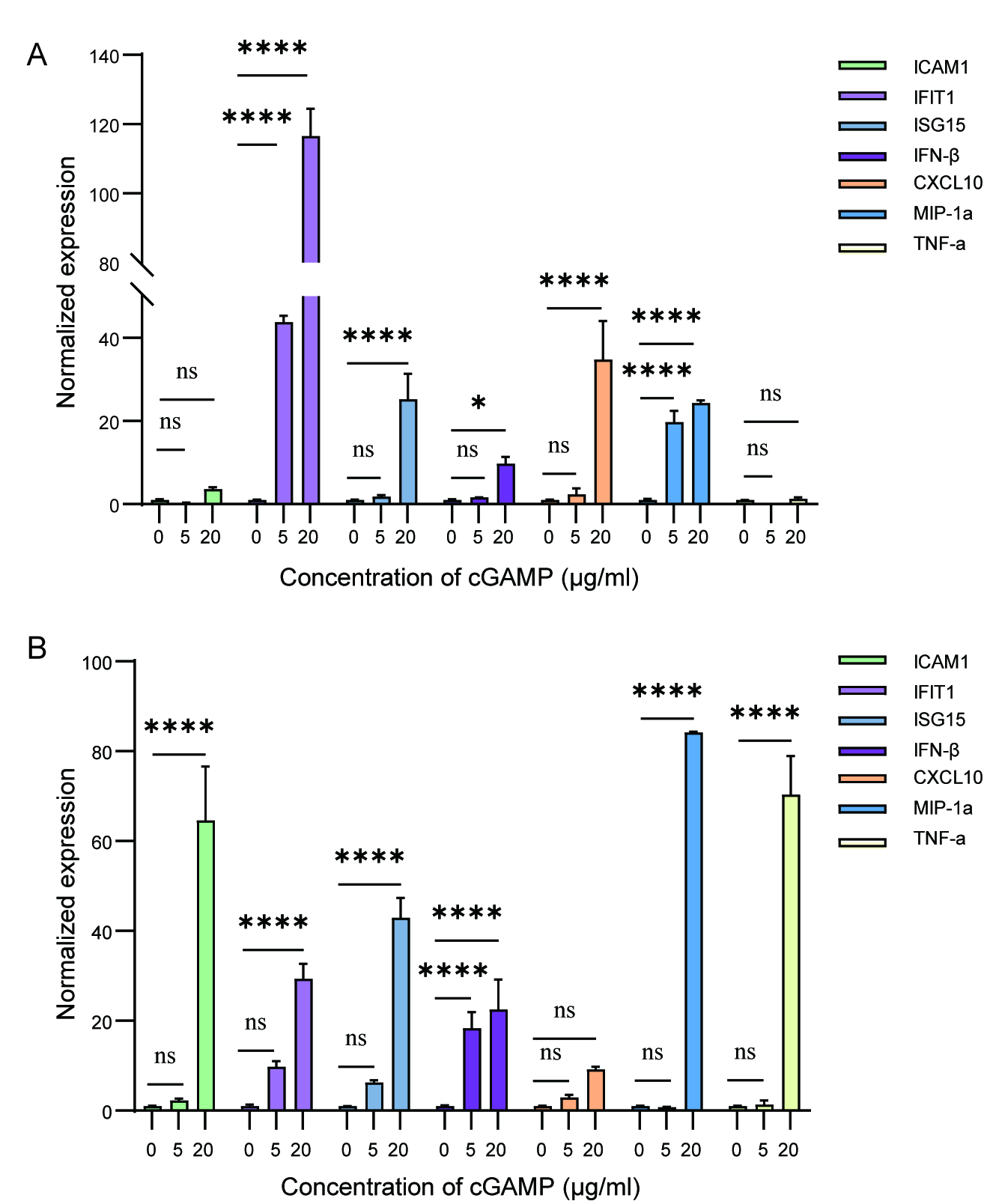


**Figure S11.** mRNA loading assay. (A-B) Luciferase mRNA was loaded into sEVs and its delivery and translation were assessed by luciferase activity. (A) Comparison of uptake efficiency between IGFBP7-modified sEVs and naive sEVs in CD93-HEK293 cells. (B) Comparison of the uptake efficiency of IGFBP7-modified sEVs between HEK293 cells and CD93-HEK293 cells. (C) mRNA loading efficiency. The copy numbers of HBVc-PEP3 mRNA in cell line and sEVs were quantified by RT-PCR. (D) HBVc-PEP3 mRNA loading and translation were evaluated by macrophage activation markers after sEVs treatment. n=3 independent samples per group, unpaired two-tailed t test. All results are expressed as means ± SD, * p < 0.05, **** p < 0.0001.

**
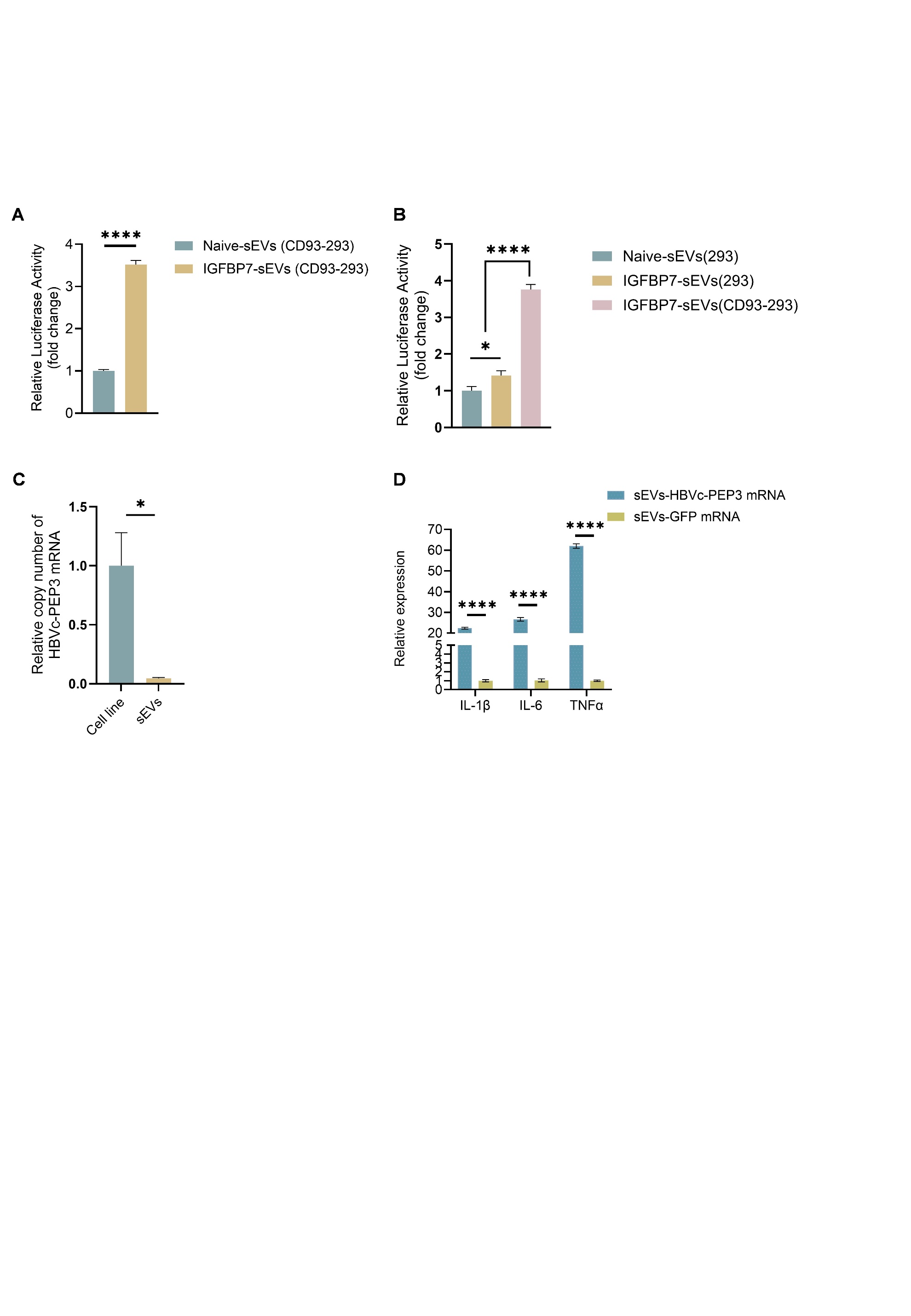
**

**Figure S12.** Evaluation of the side effect of STING agonist loaded sEVs mediated immunotherapy. (A) Mouse weight change curve. n=5 mice per group, one-way ANOVA. All results are expressed as mean ± SD, ns=not significant. (B) HE staining of peripheral tissues. Peripheral tissues from different reagents injected mice were removed and HE staining was performed to evaluate the toxicity of different reagents to the peripheral tissues. Scale bar=100 μm.


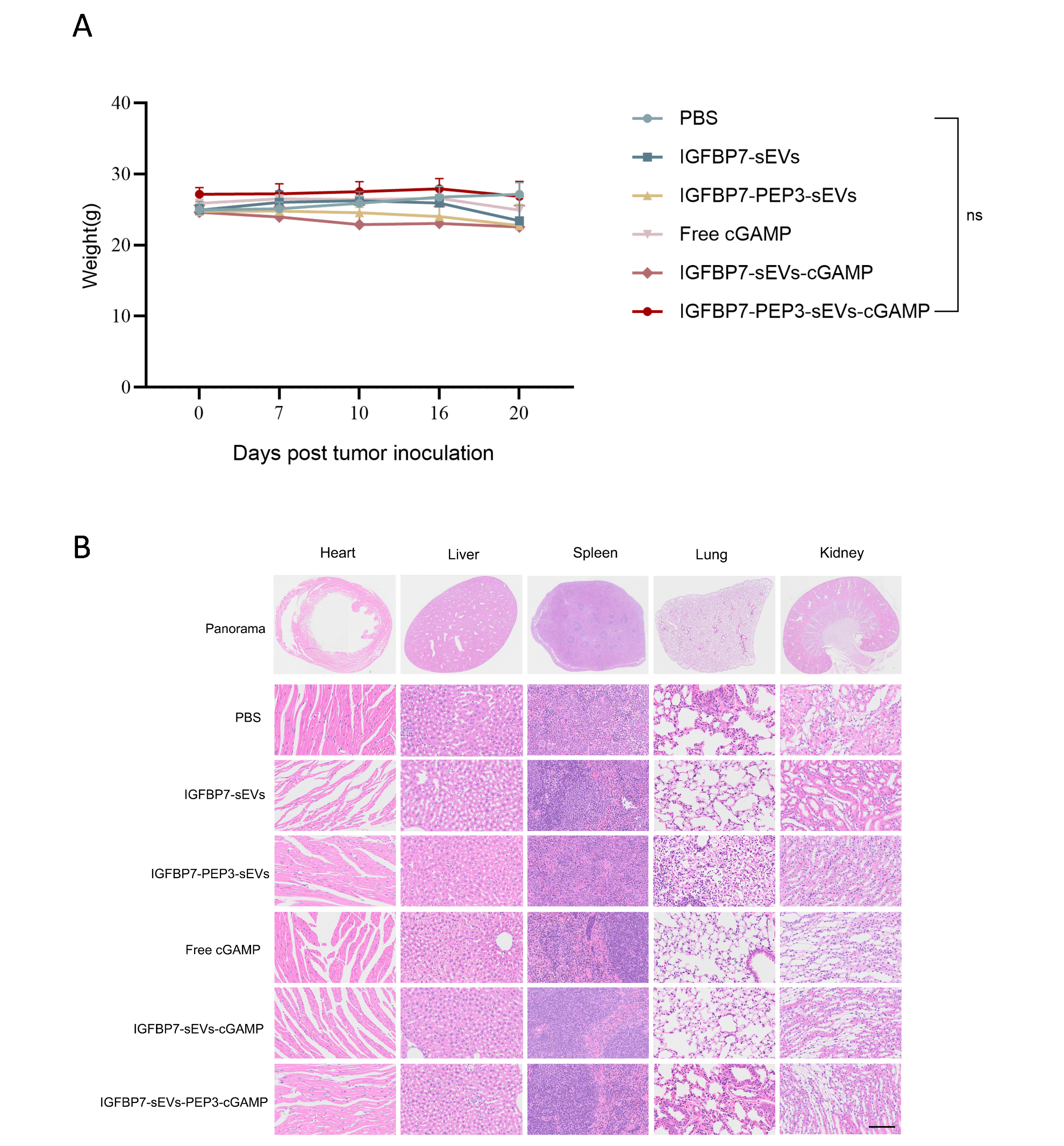


**Figure S13.** Flow cytometry analysis of tumoral infiltrated NK cells in different groups (A) and quantification of the percentage of NK cells in the tumor (B). n=5 mice per group, one-way ANOVA. Results are expressed as means ± SD, * p < 0.05, ** p < 0.01, ns= not significant.


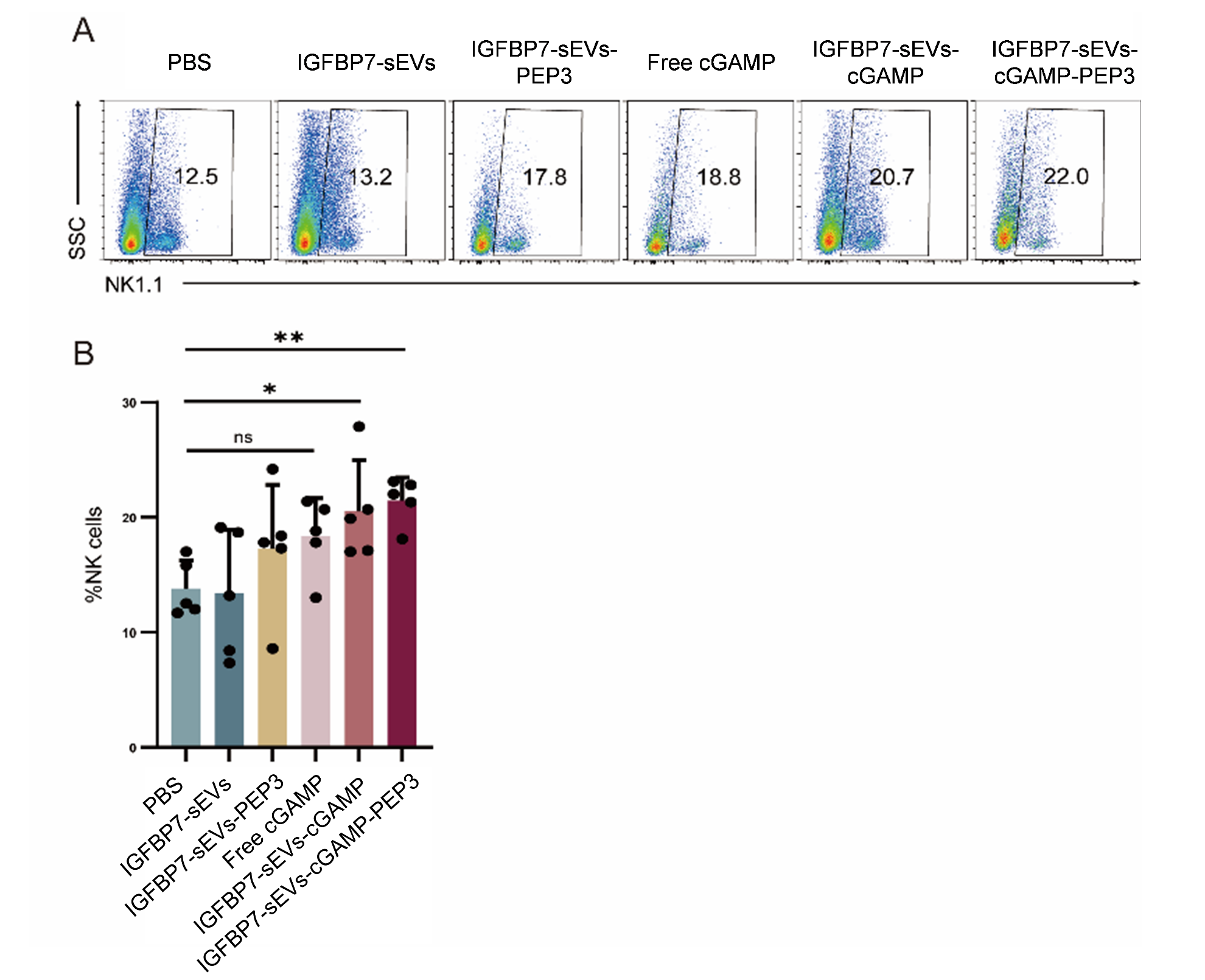


**Figure S14.** Screening of sgRNA targeted to *TMEM173* gene. (A) Schematic diagram of sgRNA targeted locations on the mouse genome. (B) T7E1 assay to verify the cleavage efficiency of different sgRNAs. Three sgRNAs targeted to mouse TMEM173 gene were synthesized and transfected into MEF-Cas9 cell line. The genomic DNAs were extracted 48 hours post transfection and the cleavage efficiency was analyzed by T7E1 assay.


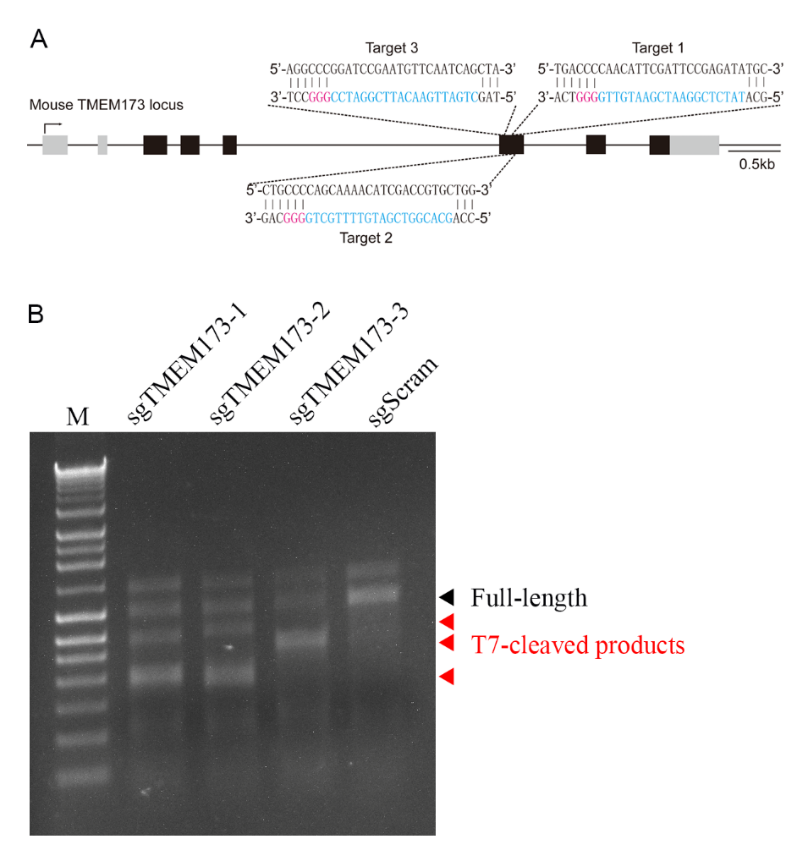


**Table S1. List of primers.**

TMEM173 sgRNA1 for: CACCgatctcggaatcgaatgttg

TMEM173 sgRNA1 reverse: AAACcaacattcgattccgagatc

TMEM173 sgRNA2 for: CACCgcacggtcgatgttttgctg

TMEM173 sgRNA2 reverse: AAACcagcaaaacatcgaccgtgc

TMEM173 sgRNA3 for: CACCgtgattgaacattcggatcc

TMEM173 sgRNA3 reverse: AAACggatccgaatgttcaatcac

TMEM173 DP for1: ctgtggtagtggtggtaggg

TMEM173 DP for2: acacacacacgggaaaacag

TMEM173 DP reverse1: aatcttgccttgtcctggga

TMEM173 DP reverse2: gcctcactcttctccaccat

TMEM173 RT For: tgagtgtagttgaccccaac

TMEM173 RT Rev: ccggcagaagagtttagcct

RT-PCR primers：

mCD93 for: TTTCCAGGCCACTACCTCCT

mCD93 reverse: CAACCAAACTTGGGGCTGAC

HBVc-PEP3 for: TACAGACCACCAAATGCCCC

HBVc-PEP3 reverse: TTGAGATCTTCTGCGACGCG

mLamp2b for: TGTCTAGAGCGTTTCAGATCAACA

mLamp2b reverse: CATCATCCAGCGAACACTCC

mICAM1 for: TCCGCTGTGCTTTGAGAACT

mICAM1 reverse: TGAGGTCCTTGCCTACTTGC

mIFIT1 for: AGTCGTAGCCTATCGCCAAG

mIFIT1 reverse: AGCTTTGGCAAGATGTGCTG

mISG15 for: GTGGTACAGAACTGCAGCGA

mISG15 reverse: TCAGCCAGAACTGGTCTTCG

mCXCL10 for : TCCATATCGATGACGGGCCA

mCXCL10 reverse: CATCGTGGCAATGATCTCAACA

mMIP-1α for: TATGGAGCTGACACCCCGAC

mMIP-1αreverse: GTCAGGAAAATGACACCTGGC

mTNF-α for: CCCTCACACTCAGATCATCTTCT

mTNF-αreverse: GCTACGACGTGGGCTACAG

mIFN-β for: CTACAGGGCGGACTTCAAGA

mIFN-β reverse: AGTCTCATTCCACCCAGTGC

mIFN-γ for: GGAACTGGCAAAAGGATGGTGA

mIFN-γ reverse: TGTTGCTGATGGCCTGATTGT

mGADPH for: TGTCAGCAATGCATCCTGCA

mGADPH reverse: GGACACATTGGGGGTAGGAACAC

mIL-1β for: GCCACCTTTTGACAGTGATGAG

mIL-1β reverse: AAGGTCCACGGGAAAGACAC

mIL-4 for: GGTCTCAACCCCCAGCTAGT

mIL-4 reverse: GCCGATGATCTCTCTCAAGTGAT

mTGF-β1 for: GCAACATGTGGAACTCTACCAGAA

mTGF-β1 reverse: GACGTCAAAAGACAGCCACTCA

**Table S2. Sequences for gene synthesis.**

1. mMMRN2

AGAATTC ggaggcggagggtcaATGCAGGACCCCGGTACCAAGTTCTCCCATCTCAATAGGCCCGGCATGCCTGAAGGCTGGAGACTAGGGGCTGAGGATACCAGCAGAGACCCCATCAGACGGAACTGGTGTCCTTACCAGAAGTCCAGGCTAGTCACCTTTGTAGCTGCTTGCAAAACAGAGAAATTCCTGGTCCATTCACAGCAGCCATGTCCACAGGGAGCCCCTGACTGCCAGGGAGTCAGAGTCATGTATCGAGTGGCCCAGAAGCCAGTGTACCAGGTCCAGCAGAAGGTGCTGATCTCTGTGGACTGGCGGTGCTGCCCAGGGTTCCAGGGACCAGACTGCCAGGACCACAATCCCACAGCAAACCCTGAGCCCACAGAGCCAAGTGGTAAACTCCAGGAGACTTGGGACTCGATGGATGGCTTTGAACTTGGTCACCCTGTCCCAGAGTTTAATGAGATTAAGGTGCCACAAGAACAACAGGAGATTCGGCGCCTGAGTTCTGATGTCAAGCAGATTGGGCAGTGCTGTGAGGCCTCCTGGGCTGCCTCCCTCAATAGCTCCCTTGAAGACCTACACAGCATGCTCTTGGACACCCAGCACGGCCTGAGACAGCACCGGCAGCTCTTCCACAACCTCTTCCAGAACTTCCAAGGTCTGGTGGCAAGCAACATCAGCCTAGACTTGGGGAAGCTGCAGGCCATGTTGAGTAAGAAAGATAAGAAGCAACCGAGAGGCCCAGGAGAATCCCGGAAGAGGGATAAGAAGCAAGTGGTGATGTCTACAGATGCACACGCCAAAGGTCTGGAGCTCTGGGAGACAGGCTCCCCTGTGGCCTTCTATGCCGGTTCTTCAGAAGGGGCCACTGCTCTGCAGATGGTGAAGTTCAACACCACATCCATCAATGTGGGCAGCAGCTACTTTCCTGAACATGGCTACTTCCGAGCTCCCAAACGTGGCGTCTACTTGTTTGCTGTGAGCATTACATTTGGCCCAGGCCCAGGAATGGGGCAGCTGGTATTTGAAGGTCATCACCGGGTTCCAGTCTACAGTACGGAACAGAGGGGCGGGAGCACAGCCACCACTTTTGCTATGGTAGAGCTACAAAAGGGTGAGAGAGCGTGGTTTGAGTTAATCCAAGGGTCAGCAACCAAAGGGAGCCAACCAGGCACTGCATTTGGGGGCTTCCTGATGTTCAAGACCggaggcggagggtcaCTCGAG T

1. mIGFBP7

AGAATTC ggaggcggagggtcaATGTCTAGCTCTGATGCTTGTGGACCTTGTGTACCTGCCAGTTGTCCAGCTCTGCCTAGGTTGGGCTGTCCACTTGGCGAAACCAGAGATGCCTGTGGCTGTTGTCCAGTTTGCGCTAGAGGCGAGGGAGAGCCTTGTGGTGGAGGCGCTGCTGGAGGTGGTCACTGTGCTCCAGGTATGGAGTGCGTGAAGTCTCGCAAACGGCGGAGAGGCAAAGCAGGTGCTGCTGCTGGCGGACCAGCTACATTGGCAGTGTGTGTGTGCAAGTCTAGGTATCCAGTGTGTGGCAGCAATGGCATCACATATCCATCTGGCTGCCAGCTGAGAGCTGCCTCTCTGAGAGCCGAAAGCAGAGGCGAGAAAGCCATCACACAGGTGAGCAAGGGAACATGCGAACAGGGACCAAGCATCGTGACACCACCTAAAGACATCTGGAACGTTACTGGTGCTAAGGTATTCTTGAGTTGTGAGGTGATTGGCATTCCAACTCCAGTGCTGATCTGGAACAAGGTGAAGAGGGATCACAGTGGTGTGCAGAGAACAGAGCTGCTGCCTGGAGATAGGGAGAACCTGGCCATCCAGACACGCGGAGGTCCAGAGAAGCACGAGGTGACAGGCTGGGTGTTGGTGAGCCCTCTGTCTAAGGAGGATGCTGGAGAGTACGAGTGCCATGCCAGCAATTCTCAGGGCCAGGCTTCTGCCGCTGCTAAGATCACCGTGGTAGATGCTCTGCATGAGATTCCACTGAAGAAGGGCGAAGGTGCTCAGCTGggaggcggagggtcaCTCGAG T

1. mCD93

TCTAGA ATGGCCATCTCAACTGGTTTGTTCCTGCTGCTGGGGCTCCTTGGCCAGCCCTGGGCAGGGGCTGCTGCTGATTCACAGGCTGTGGTGTGCGAGGGGACTGCCTGCTATACAGCCCATTGGGGCAAGCTGAGTGCCGCTGAAGCCCAGCATCGCTGCAATGAGAATGGAGGCAATCTTGCCACCGTGAAGAGTGAGGAGGAGGCCCGGCATGTTCAGCAAGCCCTGACTCAGCTCCTGAAGACCAAGGCACCCTTGGAAGCAAAGATGGGCAAATTCTGGATCGGGCTCCAGCGAGAGAAGGGCAACTGTACGTACCATGATTTGCCAATGAGGGGCTTCAGCTGGGTGGGTGGTGGAGAGGACACAGCTTATTCAAACTGGTACAAAGCCAGCAAGAGCTCCTGTATCTTTAAACGCTGTGTGTCCCTCATACTGGACCTGTCCTTGACACCTCACCCCAGCCATCTGCCCAAGTGGCATGAGAGTCCCTGTGGGACCCCCGAAGCTCCAGGTAACAGCATTGAAGGTTTCCTGTGCAAGTTCAACTTCAAAGGCATGTGTAGGCCACTGGCGCTGGGTGGTCCAGGGCGGGTGACCTATACCACCCCTTTCCAGGCCACTACCTCCTCTCTGGAGGCTGTGCCTTTTGCCTCTGTAGCCAATGTAGCTTGTGGGGATGAAGCTAAGAGTGAAACCCACTATTTCCTATGCAATGAAAAGACTCCAGGAATATTTCACTGGGGCAGCTCAGGCCCACTCTGTGTCAGCCCCAAGTTTGGTTGCAGTTTCAACAACGGGGGCTGCCAGCAGGATTGCTTCGAAGGTGGCGATGGCTCCTTCCGCTGCGGCTGCCGGCCTGGATTTCGACTGCTGGATGATCTAGTAACTTGTGCCTCCAGGAACCCCTGCAGCTCAAACCCATGCACAGGAGGTGGCATGTGCCATTCTGTACCACTCAGTGAAAACTACACTTGCCGTTGTCCCAGCGGCTACCAGCTGGACTCTAGCCAAGTGCACTGTGTGGATATAGATGAGTGCCAGGACTCCCCCTGTGCCCAGGATTGTGTCAACACTCTAGGGAGCTTCCACTGTGAATGTTGGGTTGGTTACCAACCCAGTGGCCCCAAGGAAGAGGCCTGTGAAGATGTGGATGAGTGTGCAGCTGCCAACTCGCCCTGTGCCCAAGGCTGCATCAACACTGATGGCTCTTTCTACTGCTCCTGTAAAGAGGGCTATATTGTGTCTGGGGAAGACAGTACCCAGTGTGAGGATATAGATGAGTGTTCGGACGCAAGGGGCAATCCATGTGATTCCCTGTGCTTCAACACAGATGGTTCCTTCAGGTGTGGCTGCCCGCCAGGCTGGGAGCTGGCTCCCAATGGGGTCTTTTGTAGCAGGGGCACTGTGTTTTCTGAACTACCAGCCAGGCCTCCCCAAAAGGAAGACAACGATGACAGAAAGGAGAGTACTATGCCTCCTACTGAAATGCCCAGTTCTCCTAGTGGCTCTAAGGATGTCTCCAACAGAGCACAGACAACAGGTCTCTTCGTCCAATCAGATATTCCCACTGCCTCTGTTCCACTAGAAATAGAAATCCCTAGTGAAGTATCTGATGTCTGGTTCGAGTTGGGCACATACCTCCCCACGACCTCCGGCCACAGCAAGCCGACACATGAAGATTCTGTGTCTGCACACAGTGACACCGATGGGCAGAACCTGCTTCTGTTTTACATCCTGGGGACGGTGGTGGCCATCTCACTCTTGCTGGTGCTGGCCCTAGGGATTCTCATTTATCATAAACGGAGAGCCAAGAAGGAGGAGATAAAAGAGAAGAAGCCTCAGAATGCAGCCGACAGCTATTCCTGGGTTCCAGAGCGAGCAGAGAGCCAAGCCCCGGAGAATCAGTACAGCCCAACACCAGGGACAGACTGCTGAGGATCC

**Table S3. Antibodies used in this study.**

| **Name** | **Vendor** | **Clone/** **Cat#** | **Application** | **Dilution fold** |
| --- | --- | --- | --- | --- |
| anti-mouse His tag | Servicebio | CAT#GB151251 | WB | 1:2000 |
| anti-mouse CD63 | Servicebio | CAT#GB12620 | WB | 1:500 |
| HRP-conjugated goat anti-mouse IgG | Proteintech | CAT#SA00001-1 | WB | 1:5000 |
| Rabbit anti- KI67 | Abcam | CAT#ab16667 | IHC | 1:200 |
| Rabbit anti- CD31 | Abcam | CAT#ab76533 | IHC | 1:50 |
| Goat anti- CD31 | R&D Systems | CAT#AF3628SP | IHC | 1:200 |
| Rabbit anti- pSTING | Thermo Fisher | CAT#PA5-105674 | IHC | 1:200 |
| Mouse anti- CD93 | Thermo Fisher | CAT#14-0939-82 | IHC | 1:50 |
| Chicken anti- GFP | Abcam | CAT#ab13970 | IHC | 1:2000 |
| Rabbit anti- Iba1 | Abcam | CAT# ab178846 | IHC | 1:200 |
| Rabbit anti- ARG1 | Abcam | CAT# ab203490 | IHC | 1:100 |
| Rabbit anti- iNOS | Abcam | CAT# ab115819 | IHC | 1:100 |
| Donkey anti-Mouse 488 | Abcam | CAT#ab150105 | IHC | 1:500 |
| Donkey anti-Mouse 594 | Abcam | CAT#ab150108 | IHC | 1:500 |
| Donkey anti-Rabbit 488 | Abcam | CAT#ab150073 | IHC | 1:500 |
| Donkey anti-Rabbit 594 | Abcam | CAT#ab150076 | IHC | 1:500 |
| Donkey anti-Goat 594 | Jackson ImmunoResearch Laboratories | CAT#705-585-147 | IHC | 1:500 |
| Donkey anti-Chicken 488 | Jackson ImmunoResearch Laboratories | CAT#703-545-155 | IHC | 1:500 |
| Mouse CD45 PerCP-Cy5.5 | BioLegend | 30-F11  CAT# #103132 | FC | 1:100 |
| Mouse CD45 FITC | eBioscience | 30-F11  CAT#11-0451-85 | FC | 1:100 |
| Mouse CD3 PerCP-Cy5.5 | BioLegend | 145-2C11 CAT#100327 | FC | 1:100 |
| Mouse CD4 APC | eBioscience | RM4-5  CAT#17-0042-82 | FC | 1:100 |
| Mouse CD8a APC/Cy7 | BioLegend | 53-6.7  CAT#100708 | FC | 1:100 |
| Mouse NK1.1 PE-Cy7 | eBioscience | PK136  CAT#25-5941-82 | FC | 1:100 |
| Mouse PD-1 PE/Dazzle™ 594 | BioLegend | 29F.1A12  CAT#135227 | FC | 1:100 |
| Mouse PD-1 PE | BioLegend | 29F.1A12  CAT#135205 | FC | 1:100 |
| Mouse CD11b APC/Cy7 | BioLegend | clone M1-70  CAT#101226 | FC | 1:100 |
| Mouse MHCII PE-EF610 | eBioscience | M5/114.15.2 , CAT#416-5321-82 | FC | 1:100 |
| Mouse IFN-γ PE | BioLegend | XMG1.2  CAT#505807 | FC | 1:100 |
| Mouse IFN-γ APC | BioLegend | XMG1.2 CAT#505810 | FC | 1:100 |
| Mouse IL-10 APC | BioLegend | JES5-16E3  CAT#505009 | FC | 1:100 |
| Mouse TNF-α PE-Cy7 | eBioscience | MP6-XT22  CAT#25-7321-80 | FC | 1:100 |
